# Beyond light scattering: the effects of intralipid on benzoporphyrin derivative-sensitized photodynamic treatment in ovarian cancer cells

**DOI:** 10.1117/1.JBO.30.S3.S34116

**Published:** 2025-12-22

**Authors:** Marta Overchuk, Albert M. Choi, Gavin A. E. Wiltshire, Huang-Chiao Huang, Albert W. Girotti, Imran Rizvi

**Affiliations:** a University of North Carolina at Chapel Hill, North Carolina State University, Lampe Joint Department of Biomedical Engineering, Chapel Hill & Raleigh, North Carolina, United States; b University of Maryland, Fischell Department of Bioengineering, College Park, Maryland, United States; c University of Maryland School of Medicine, Marlene and Stewart Greenebaum Cancer Center, Baltimore, Maryland, United States; d Medical College of Wisconsin, Department of Biochemistry, Milwaukee, Wisconsin, United States

**Keywords:** Intralipid, photodynamic therapy, lipid peroxidation, ovarian cancer

## Abstract

**Significance:**

Intralipid, a soybean oil-based lipid emulsion, is widely used in photomedicine to enhance light distribution due to its strong scattering properties. Although the optical characteristics of Intralipid are well documented, interactions with the reactive molecular species (RMS) generated during photodynamic therapy (PDT) and the impact of such interactions on therapeutic outcomes remain poorly understood. We reveal that Intralipid actively influences PDT response *in vitro*, beyond its role as a scattering agent.

**Aim:**

We examined how Intralipid affects the optical and photodynamic behavior of benzoporphyrin derivative (BPD), a clinical photosensitizer, in solution and across four ovarian cancer cell lines.

**Approach:**

The photodynamic properties of BPD, with and without Intralipid, were analyzed using fluorescence spectrometry and RMS probes, and PDT-induced oxidation of Intralipid components was characterized using LC-MS. The effects of Intralipid on BPD-PDT were evaluated under various conditions.

**Results:**

Intralipid reduced BPD photobleaching and RMS generation, suggesting RMS quenching. Extensive oxidation of Intralipid components was observed following PDT. Finally, Intralipid significantly modified BPD-PDT efficacy across all four cell lines, depending on photosensitizer–light interval, dose, and incubation time.

**Conclusions:**

Intralipid acts as a bioactive modulator of PDT response, highlighting the need for further investigations both *in vitro* and *in vivo*.

## 1 Introduction

Intralipid, a milky-white fat emulsion, is widely utilized in parenteral nutrition to supply essential calories and fatty acids.^1^^,^^2^ It is primarily composed of soybean oil, egg yolk phospholipids, and glycerol, forming ∼500  nm droplets in aqueous suspension, typically supplied as a 20% solution.^3^ Due to its high scattering and low absorption coefficients, Intralipid has long been employed in tissue phantom studies and as a scattering agent in photodynamic therapy (PDT).^4^^,^^5^ PDT utilizes a photosensitizer and visible light to produce cytotoxic reactive molecular species (RMS).^6^^,^^7^ Dilute Intralipid solutions can help achieve homogeneous light fluences, a critical factor when dealing with complex irradiation geometries,^8^^,^^9^ an example of which is the use of Intralipid in PDT for intraperitoneal metastases, such as those found in ovarian cancer.

The vast majority (over 80%) of patients diagnosed with high-grade serous ovarian carcinoma (HGSOC), the most prevalent form of ovarian cancer, present with metastatic disease.^10^^–^^13^ Despite significant advancements across basic, translational, and clinical research, the dismal ∼30% 5-year survival rate for HGSOC has remained largely unchanged for decades.^14^^–^^16^ Curative outcomes are rarely achieved largely because complete resection of tumor deposits is inherently challenging, given their common spread across the peritoneal wall, mesentery, and omenta.^17^ In this context, PDT has emerged as a promising investigational approach. Numerous preclinical studies have explored PDT as a strategy to target residual tumor cells post-surgery.^8^^,^^18^^–^^22^ These investigations have examined PDT as both a standalone modality and, more recently, as a “priming” strategy to sensitize target cells to adjuvant treatments.^20^^,^^23^^–^^29^ A frequently cited characteristic of PDT—the limited depth of penetration of visible light—may, in fact, confer a distinct advantage in this specific clinical scenario.^9^^,^^30^ Provided the photosensitizer exhibits a sufficient tumor-to-tissue accumulation ratio,^31^ PDT can effectively target shallow tumor deposits (typically <1  cm in depth) that persist after surgical resection while sparing underlying healthy tissues.

To harness these promising characteristics of PDT, significant research efforts have been devoted to intraoperative light delivery strategies.^8^^,^^9^^,^^32^ Among the goals of these efforts is to ensure that adequate fluences are delivered to all relevant metastatic sites. Within this context, intraperitoneal administration of Intralipid has been shown to improve light distribution, thereby enhancing both the uniformity of coverage and the depth of light penetration. For instance, Lilge et al.^32^ examined the impact of Intralipid on intraperitoneal light distribution and PDT efficacy in athymic nude mice bearing intraperitoneal OVCAR-3 tumors and injected with either 2 mL of phosphate-buffered saline (PBS) or Intralipid (2% or 0.2% w/v). Intralipid enabled a more homogeneous illumination of the peritoneum than PBS, thereby enhancing the activation of the photosensitizer, particularly in areas adjacent to the highly light-absorbing liver, as demonstrated by careful *in situ* light dosimetry measurements. Furthermore, the injection of 0.2%, but not 2% Intralipid, resulted in a notable increase in light penetration depth, from 1.6 to 2.3 mm.

Given the strong preclinical evidence,^8^^,^^32^ intraperitoneal administration of 0.01% to 1% Intralipid has been adopted clinically for patients with recurrent ovarian carcinomatosis or peritoneal sarcomatosis.^9^^,^^33^^–^^37^ In a phase I trial, patients received intravenous Porfimer sodium, a first-generation photosensitizer, before undergoing laparotomy and cytoreductive surgery, with the aim of debulking tumor deposits to <5  mm in thickness.^33^^,^^34^ Following irradiation of the mesentery and bowel using flat-cut optical fibers, the remaining peritoneal cavity was filled with an Intralipid solution and treated with a light-diffusing wand. Since these early studies, numerous groups have implemented the use of Intralipid to improve light distribution during PDT for various cancers, including those of the pleura,^38^^–^^40^ bladder,^41^^,^^42^ and brain.^39^^,^^43^

Although the optical properties of Intralipid, such as light scattering and attenuation, are well established, its influence on the photochemical behavior of dissolved photosensitizers and its own noninert chemical nature are increasingly recognized.^3^^,^^44^^–^^46^ Recent studies highlighted a significant influence of Intralipid on the RMS produced during photochemical reactions, including both type I (electron transfer) and type II (energy transfer to oxygen, producing singlet oxygen, O12) mechanisms.^47^ For instance, Vikas et al.^46^ utilized time-resolved O12 luminescence detection to demonstrate that Intralipid substantially quenches O12 produced by protoporphyrin IX in various media, including ethanol, acetone, and biologically relevant solutions. Beyond altering PDT-induced RMS generation, Intralipid has also been hypothesized to trap RMS through oxidation. This is primarily attributed to the presence of oxidation-sensitive unsaturated lipid groups (e.g., oleic, linoleic, and linolenic acids) in the soybean oil component. Supporting this, Durantini et al. showed that Intralipid components interact with photochemically generated RMS, suggesting the possibility of photooxidation reactions involving Intralipid through O12 “ene” reactions and [2+2] cycloadditions, as well as direct type I reactions.^3^^,^^48^^–^^50^

These Intralipid-RMS interactions are highly significant for PDT applications. The quenching of O12 could directly alter PDT outcomes, potentially reducing cytotoxic efficacy. Furthermore, the implications of oxidation products of Intralipid on cancer cells and surrounding healthy tissues remain largely unknown. Hence, the present study begins to explore these critical questions by establishing the effects of Intralipid on the optical properties and PDT efficacy of benzoporphyrin derivative (BPD), a clinically approved photosensitizer, both in solution and across four ovarian cancer cell lines (OVCAR-3, Caov-3, OVCAR-8, and OVCAR-5). Furthermore, here, we present, for the first time to our knowledge, a liquid chromatography–mass spectrometry (LC-MS) analysis of Intralipid components following exposure to BPD-PDT.

## 2 Materials and Methods

### 2.1 In Solution Studies

#### 2.1.1 Intralipid filtration

Intralipid (20%, Baxter Healthcare, Deerfield, Illinois, United States) was diluted in PBS (Corning, Cat. No. 21-040-CM, Corning, New York, United States) to prepare 2 mL of 1% Intralipid intermediate solution. A Pall nylon syringe filter (Cat. No. PN4433, Port Washington, New York, United States) was pre-wetted with PBS, after which 1 mL of 1% Intralipid solution was passed through the filter (Fig. S1 in the Supplementary Material). Filtered and unfiltered Intralipid solutions were then further diluted in PBS to final concentrations ranging from 0 to 0.1% Intralipid. Each sample was transferred into a white-walled, clear-bottom 96-well plate (Corning, Cat. No. 3610) in triplicate at 200  μL/well. Absorbance spectra (400 to 800 nm) and optical density at 687 nm were measured using a CLARIOstar Plus plate reader (BMG LABTECH, Cary, North Carolina, United States).

#### 2.1.2 UV–Vis absorbance

A 0.001% Intralipid solution was prepared by diluting a 1% Intralipid stock with PBS. BPD (≥94% HPLC, Sigma Aldrich, Cat. No. SML0534, St. Louis, Missouri, United States) solutions (0 to 10  μM) were prepared in either PBS or 0.001% Intralipid. The absorbance spectra of BPD (400 to 800 nm) were collected using a CLARIOStar Plus plate reader. Baseline correction was performed by subtracting the absorbance of the corresponding PBS or 0.001% Intralipid blank from each BPD spectrum.

#### 2.1.3 Fluorescence emission and quenching

Triton X-100 (Sigma, Cat. No. X100-100ML) was diluted in PBS to produce a 1% v/v solution. The concentration of BPD in dimethylsulfoxide (DMSO, cell culture grade, ATCC) stocks was verified using a UV–Vis spectrophotometer (Cary 60 UV-Vis, Agilent, Santa Clara, California, United States). Working solutions containing 1  μM BPD were prepared in either 1% Triton X-100 or 0-0.1% Intralipid. Aliquots (200  μL) of each solution were transferred in triplicate to a white-walled, clear-bottom 96-well plate. BPD fluorescence in PBS and Triton X-100 was measured using a CLARIOstar Plus plate reader at Ex/Em: 435-10/700-100 nm. Quenching efficiency of BPD was calculated using the following formula, where $F_{\mathrm{PBS}}$ represents BPD fluorescence in PBS and $F_{\mathrm{TX}}$ represents BPD fluorescence in Triton X-100:

$$\text{Quenching efficiency},\;\%=(1-\frac{F_{\mathrm{PBS}}}{F_{\mathrm{TX}}})\times 100$$

#### 2.1.4 Effects of intralipid on RMS generation of BPD

BPD solutions (1  μM) were prepared in PBS containing 0% to 0.1% Intralipid from DMSO stocks. These solutions were then transferred to white-walled, clear-bottom 96-well plates at 0.18  mL/well.

Fresh singlet oxygen sensor green (SOSG, Invitrogen, Cat. No. S36002, Carlsbad, California, United States) stock was prepared by dissolving 100  μg of SOSG in 33  μL of methanol, yielding a 5 mM stock, which was then diluted to 100  μM with PBS. Hydroxyphenyl fluorescein (HPF, Molecular Probes, Cat. No. H36004, Eugene, Oregon, United States), supplied as a 5 mM solution in dimethylformamide, was diluted with PBS to prepare a 200  μM stock solution. To each well containing 0.18 mL of BPD ± Intralipid solution, 20  μL of SOSG or HPF was added, yielding final concentrations of 10 and 20  μM, respectively. The plates were irradiated using a 690 nm LED irradiation platform (LEDBox, BioLambda, São Paulo, Brazil) at an irradiance of $20.04\;\mathrm{mW}/{\mathrm{cm}}^{2}$ and escalating energy densities between 0 and $5\;J/{\mathrm{cm}}^{2}$. Irradiation times were automatically calculated by a controller (BlackBox Smart, BioLambda) to deliver the desired energy densities. SOSG and HPF fluorescence intensities were measured using a CLARIOstar Plus plate reader at Ex/Em: 495-8/525-8 and 485-10/530-30, respectively. Fluorescence values of SOSG and HPF in BPD ± Intralipid solutions at $0\;J/{\mathrm{cm}}^{2}$ were subtracted as a background. Background-corrected values were then divided by BPD fluorescence intensity at corresponding energy densities to account for BPD photobleaching. Nonlinear regressions were derived in Graphmatik (all $R^{2}\text{ values}>0.95$).

#### 2.1.5 Lipid radical generation

C11 BODIPY581/591 (Sigma-Aldrich, SML3717) was dissolved in DMSO at 2 mM and stored at −20°C. A 100  μM intermediate stock was prepared by diluting the DMSO stock in PBS. Working solutions containing 1  μM BPD in PBS containing 0% to 0.1% Intralipid were prepared as described above. For each assay, 20  μL of C11 BODIPY was added to wells of a white-walled 96-well plate containing 180  μL of the BPD ± Intralipid solutions for a final concentration of 10  μM. A second, identical plate was prepared using C11 BODIPY in PBS with 0% to 0.1% Intralipid, but without BPD. Using the LED irradiation platform, both plates were exposed to 690 nm light at an irradiance of $20.04\;{\mathrm{mW}/\mathrm{cm}}^{2}$ and doses of 0, 0.5, 1, 2.5, and $5\;J/cm^{2}$. Fluorescence was measured using a CLARIOStar Plus plate reader at 480 to 10 nm excitation and 530 to 20 nm emission (oxidized form), and 560 to 15 nm excitation and 600 to 15 nm emission (unoxidized form). Oxidized/total C11 BODIPY fluorescence ratios were calculated and normalized to the $0\;J/cm^{2}$ wells for each Intralipid concentration.

### 2.2 Lipidomic Analysis

To investigate the effects of BPD-PDT on Intralipid chemical composition, 1  μM BPD solutions in PBS containing 0.1% Intralipid were either protected from light or exposed to $5\;J/{\mathrm{cm}}^{2}$ of 690 nm light at $20.04\;\mathrm{mW}/{\mathrm{cm}}^{2}$ using the LED irradiation platform described above. Both dark controls and light-irradiated BPD-Intralipid solutions (2 mL per sample) were concentrated in a SpeedVac vacuum concentrator at the lowest temperature setting overnight, after which the pellets were subjected to lipidomic workflows established at the Department of Chemistry Mass Spectrometry Core Laboratory at the University of North Carolina at Chapel Hill. Briefly, 1 mL of methyl tert-butyl ether (MTBE) was added to the pellets, vortexed, and then transferred to 1.5 mL centrifuge tubes. Then, 300  μL of 1.5  μg/mL of EquiSPLASH (Avanti Research, Alabaster, Alabama, United States) in methanol (internal standard) was added, and the samples were shaken for 10 min. Next, 200  μL of water was added to facilitate phase separation, and the extracts were centrifuged at 20,000 rcf for 10 min. The top layer was collected, dried down, and reconstituted in 100  μL of isopropyl (IPA) alcohol for analysis. Lipidomic analysis was performed using a Thermo Q Exactive HFX coupled to a Waters Acquity H-Class LC. A 100×2.1  mm, 1.7  μm Waters BEH C18 column was used for separations. The following mobile phases were used: A- 60/40 acetonitrile (ACN)/H2O, B- 90/10 IPA/ACN; both mobile phases had 10 mM ammonium formate and 0.1% formic acid. A flow rate of 0.2  mL/min was used. The starting composition was 40% B, which increased to 95% B at 12 min (held until 15 min) then 40% B at 15.1 min and held for 5 min for re-equilibration. Samples were analyzed in positive/negative switching ionization mode with the top five data-dependent fragmentation. Raw data were analyzed by LipidSearch software (Thermo Fisher Scientific, Waltham, Massachusetts, United States). Lipids were identified by MS2 fragmentation (mass error of precursor = 5 ppm, mass error of product = 8 ppm). The identifications were generated individually for each sample and then aligned by grouping the samples (OxPAPC = C, HF = S1, Con = S2). Normalization was performed using EquiSPLASH from Avanti.

From 2631 lipids identified by LipidSearch, 1020 adducts and 29 internal standards were excluded. The remaining dataset comprised 993 “Unoxidized” lipid IDs and 589 “Oxidized” lipids (containing ─OH or ─OOH groups). The sum peak areas for each lipid class were calculated to assess class-level changes in oxidized and unoxidized states. Changes in the abundances of all oxidized phosphatidylcholines (oxPC), phosphatidylethanolamines (oxPE), and triglycerides (oxTGs) were assessed by calculating Z-scores and visualized as heatmaps (available in the Supplementary Material). For detailed species-level analysis, lipids showing an absolute fold change >1 and p<0.05 were selected. Their chromatograms were manually reviewed in LipidSearch software (Thermo Fisher Scientific) to remove duplicates and exclude erroneously integrated peaks. Sample identities were blinded during this process, and evaluations were based solely on peak quality metrics, including shape and retention time reproducibility. Of the 438 lipids showing significant changes post-PDT, 297 passed this review (Table S1 in the Supplementary Material). Log2 fold changes of the select oxPCs, oxPEs, and oxTGs were visualized using volcano plots and heatmaps.

### 2.3 Cell Culture

Human epithelial ovarian adenocarcinoma OVCAR-3, Caov-3, OVCAR-5, and OVCAR-8 cells were obtained from the American Type Culture Collection (ATCC). OVCAR-3 cells were grown in RPMI 1640 medium (Gibco, Thermo Fisher Scientific) supplemented with 20% heat-inactivated fetal bovine serum (FBS, Cytiva, lot# AK30785350, Marlborough, Massachusetts, United States), 0.01  mg/mL recombinant human insulin (Gibco, Thermo Fisher Scientific), 100  U/mL penicillin, and 100  μg/mL streptomycin (Sigma-Aldrich, St. Louis, MO). Caov-3 cells were grown in Dulbecco’s Modified Eagle’s Medium High Glucose (DMEM, Sigma-Aldrich) supplemented with 10% FBS, 100  U/mL penicillin, and 100  μg/mL streptomycin. OVCAR-5 and OVCAR-8 cells were grown in RPMI 1640 medium supplemented with 10% FBS and 100  U/mL penicillin and 100  μg/mL streptomycin. Cells were maintained in 2D monolayers at 37°C in a humidified incubator with 5% ${\mathrm{CO}}_{2}$ and routinely tested for *Mycoplasma* contamination using the MycoAlertTM PLUS Kit (Lonza Bioscience, Cat. No. LT07-710, Basel, Switzerland). Cells were propagated until they reached passage 30.

### 2.4 Dark Toxicity of Intralipid

Cells were seeded in white-walled, clear-bottom 96-well plates at densities optimized for the CellTiter-Glo linear range at 120 h: OVCAR-3 $-5\times 10^{3}$, Caov-3 $-1\times 10^{4}$, OVCAR-8, and OVCAR-5 $-1.25\times 10^{3}\text{ cells}$ per well. After 48 h of incubation, wells were treated with Intralipid at concentrations of 0%, 0.001%, 0.01%, and 0.1%. A 2% intermediate Intralipid solution was prepared in sterile PBS from the 20% stock, filtered through a pre-wetted Pall nylon syringe filter (Cat # PN 443), and diluted in complete cell culture media to the desired concentrations. After 72 h of treatment, Intralipid solutions were removed, and cell viability was assessed using the CellTiter-Glo assay.

### 2.5 Photodynamic Therapy

For short drug-light interval PDT (SDLI-PDT), cells were seeded in white-walled, clear-bottom 96-well plates at the densities described above. After 48 h, the culture medium was replaced with complete medium containing BPD at 0.125, 0.25, or 1  μM, and Intralipid at 0%, 0.001%, or 0.01%. Immediately following BPD addition, cells were irradiated with 690 nm light at doses of 0, 0.03, 0.05, 0.1, 0.25, 0.5, and $1\;J/{\mathrm{cm}}^{2}$, using the LED irradiation platform. After irradiation, the BPD-Intralipid solutions were either left in the wells or replaced with fresh complete medium. For conventional PDT, cells were first incubated with 0.125  μM BPD for 90 min. The BPD-containing medium was then removed and replaced with 200  μL of complete medium containing 0%, 0.001%, or 0.01% Intralipid. Plates were irradiated under the same conditions as in SDLI-PDT. Following irradiation, the Intralipid-containing medium was either left in place or replaced with fresh complete medium. In all conditions, cell survival fractions were measured 72 h post-PDT.

### 2.6 Cytotoxic Effects of PDT-Exposed Intralipid

Cells were seeded in one white-walled, clear-bottom 96-well plate per cell line at cell densities optimized for the CellTiter-Glo linear range at 120 h: OVCAR-3 $-5\times 10^{3}$, Caov-3 $-1\times 10^{4}$, OVCAR-8, and OVCAR-5 $-1.25\times 10^{3}\text{ cells}$ per well. After 48 h of incubation, a 2% intermediate Intralipid solution was prepared from the 20% stock in sterile PBS, filtered through a Pall nylon syringe filter, pre-wetted with PBS, and diluted in complete cell culture media. Four treatment conditions were then prepared for each cell line: complete medium only (medium control), complete medium with 1  μM BPD (BPD only), complete medium with 0.01% Intralipid (Intralipid only), and complete medium with 1  μM BPD and 0.01% Intralipid (Intralipid + BPD). Each solution was dispensed at 250  μL per well in a separate cell-free white-walled, clear-bottom 96-well plate, corresponding to each cell line, and irradiated from the bottom using the 690 nm LED irradiation platform at $20.04\;\mathrm{mW}/cm^{2}$, delivering energy densities of 0, 0.1, 0.5, 1, 5, and $10\;J/{\mathrm{cm}}^{2}$. Each energy density was delivered sequentially, after which the old media were removed from the cell-containing plates, and 200  μL of the corresponding irradiated treatment solution was transferred to each well. Medium control and Intralipid + BPD combination conditions included three technical replicates, whereas BPD only and Intralipid only conditions included two technical replicates. After 72 h of treatment, all dosing solutions were removed, and cell viability was assessed using the Cell Titer-Glo assay. Survival fractions were calculated by normalizing each well’s luminescence to the average value of the medium-only controls on the same plate.

### 2.7 Survival Fraction Measurements

Cells in 96-well plates were subjected to various treatments, including different concentrations of Intralipid, BPD, light, and their combinations. Cell survival fractions were assessed using the CellTiter-Glo Luminescent Cell Viability Assay (Catalog #C7572, Promega Corp., Madison, Wisconsin, United States). Briefly, at the designated endpoint, the culture medium in each well was removed and replaced with 50  μL of fresh medium. Following the manufacturer’s instructions, the plates were equilibrated at room temperature for 30 min, after which 50  μL of CellTiter-Glo reagent was added to each well. Plates were agitated for 2 min (orbital shaking, 300 rpm), followed by a 10-min incubation at room temperature. Luminescence was measured using a CLARIOstar Plus plate reader. Luminescence values from treated wells were normalized to those from untreated control wells on the same plate, where cells were exposed only to complete cell culture media. Cell seeding densities for Intralipid dark toxicity and BPD-PDT experiments were optimized based on the linear dynamic range of the CellTiter-Glo Assay (Figs. S2 and S3 in the Supplementary Material).^24^

### 2.8 Statistical Analysis and Data Visualization

Each study was performed in three or more independent repeats containing at least two technical replicates. For solution-based assays, an independent repeat refers to a trial conducted on a different day using freshly prepared working solutions of all reagents. For cell-based assays, an independent repeat is defined as one performed using a different cell passage and freshly prepared working solutions. In each case, technical replicates refer to parallel measurements or samples processed within the same experimental run (e.g., different wells in a 96-well plate). Intralipid working solutions were freshly diluted from the same 20% stock for each independent experiment. BPD working solutions were prepared from aliquots of a single ∼500  μM stock, and the BPD concentration in each aliquot was verified prior to use in every independent experiment.

Unpaired t-tests and one-way or two-way ANOVAs were employed as specified in figure captions using Graphmatik.io, a web-based visualization platform (version 0.3.2, 2025) or GraphPad Prism (version 10.4.0). Dunnett or Šidák corrections for multiple comparisons were used in one-way and two-way ANOVAs, respectively, as specified in figure captions. All tests are 2-sided using an alpha level of 0.05. To generate heatmaps, fold changes, p-values, and Z-scores were calculated using R (version 4.4.1) and plotted using the ggplot2 package.

## 3 Results and Discussion

### 3.1 Effects of Intralipid on BPD Properties

Prior to investigating the effects of Intralipid on BPD-PDT, we characterized changes in BPD fluorescence, photobleaching, and RMS generation in the presence of Intralipid in aqueous solutions.

#### 3.1.1 Absorbance spectrum

**Fig. 1:**
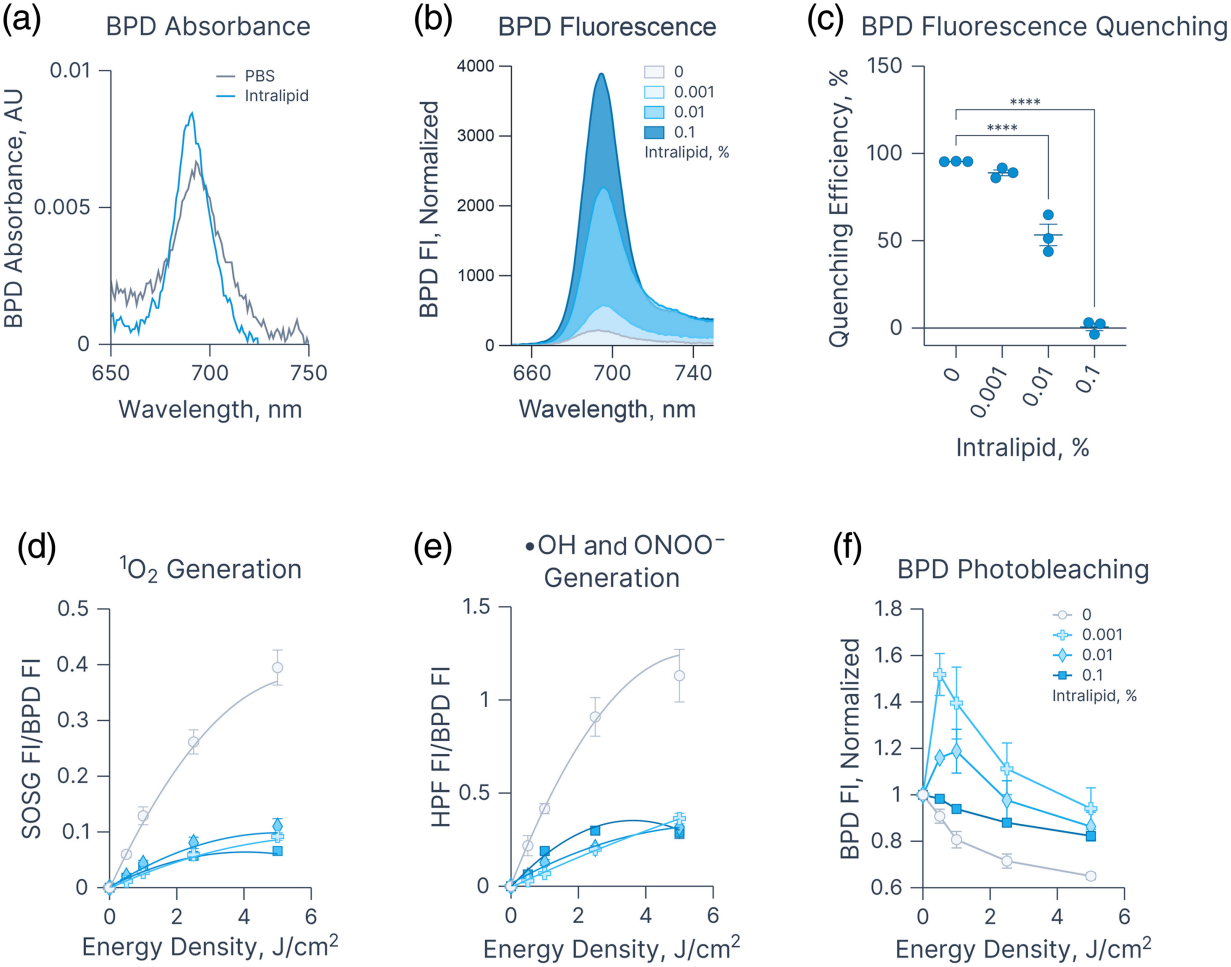
Effects of Intralipid on the optical properties and photodynamic RMS generation of BPD. (a) Absorbance and (b) fluorescence emission spectra of 1  μM BPD. (c) Aggregation-induced fluorescence quenching of BPD. Photodynamically generated (d) singlet oxygen and (e) hydroxyl radical/peroxynitrite. (f) Photobleaching of BPD following $0\text{–}5\;J/{\mathrm{cm}}^{2}$ of 690 nm light exposure. Statistics: (a), (b) Mean absorbance and fluorescence emission spectra from three independent repeats each conducted in duplicate (technical replicates). (c) Each point denotes the mean of an independent repeat (each performed in duplicate). (d)–(f) Each point represents the mean of three independent repeats each conducted in duplicate (technical replicates). Lines in panels (d) and (e) represent simple polynomial regression fits. All central tendencies are means, with error bars indicating the standard error of the mean. All graphs were generated using Graphmatik.

We examined the impact of Intralipid on the absorbance characteristics of BPD. To minimize scattering effects, a dilute Intralipid solution (0.001%) was employed. In the presence of Intralipid, the $Q_{y}$ band of 1  μM BPD in PBS exhibited a pronounced blue shift, with the peak moving from 694 to 691 nm [Fig. 1(a)]. A similar shift was observed at 2.5 and 5  μM BPD; however, at 10  μM, the addition of Intralipid failed to induce this spectral change, suggesting a concentration-dependent threshold (Fig. S4 in the Supplementary Material). We hypothesized that this blue shift reflects Intralipid-mediated dissociation of BPD aggregates, which may in turn reduce aggregation-induced fluorescence quenching.

#### 3.1.2 Fluorescence emission

The addition of Intralipid to a 1  μM BPD solution in PBS resulted in a concentration-dependent enhancement of BPD fluorescence [Fig. 1(b)]. To further characterize the effect of Intralipid on aggregation-induced quenching, we introduced Triton X-100 as a detergent control. Although BPD fluorescence was strongly quenched in PBS alone (95.36±0.08%), the inclusion of 0.001%, 0.01%, and 0.1% Intralipid progressively reduced quenching to 88.87±1.63%, 53.31±6.15%, and 0.62±2.23% respectively [Fig. 1(c), Fig. S5 in the Supplementary Material]. These findings suggest that the fluorescence enhancement observed in Intralipid-containing solutions is attributable to increased BPD solubilization within lipid droplets, leading to reduced self-quenching from aggregation.

#### 3.1.3 Photochemical generation of RMS

Next, we investigated the effects of Intralipid on the generation of RMS using two fluorescence sensors: SOSG, selective for O12, and HPF,^51^ selective for hydroxyl radical and peroxynitrite. BPD in PBS containing 0%, 0.001%, 0.01%, and 0.1% Intralipid was exposed to 0 to $5\;J/{\mathrm{cm}}^{2}$ of 690 nm light in the presence of 10  μM SOSG or 20  μM HPF. Interestingly, raw fluorescence intensity values of SOSG and HPF were significantly higher in BPD-Intralipid solutions compared with BPD in PBS upon light exposure, but not in the dark (Figs. S4–S8 in the Supplementary Material). To account for differences in aggregation-induced quenching of BPD, SOSG and HPF fluorescence intensities were normalized to BPD fluorescence in their respective Intralipid concentrations, which revealed a relative decrease in RMS generation with increasing Intralipid concentration [Figs. 1(d) and 1(e)].

#### 3.1.4 Photobleaching

Next, the impact of Intralipid on BPD photobleaching was assessed [Fig. 1(f)]. Irradiation of a 1  μM BPD solution in PBS with $5\;J/{\mathrm{cm}}^{2}$ of 690 nm light reduced BPD fluorescence to 0.65±0.01 of its initial value. The addition of Intralipid at 0.001%, 0.01%, and 0.1% markedly slowed the rate of BPD photobleaching, resulting in higher fluorescence signals compared with the BPD solution in PBS alone after exposure to $5\;J/{\mathrm{cm}}^{2}$ (0.94±0.09, 0.86±0.06, and 0.82±0.02 of the initial BPD fluorescence, respectively). Interestingly, at 0.001% and 0.01% Intralipid, an initial increase in fluorescence was observed at lower energy densities, likely resulting from BPD unquenching and redistribution between lipid droplets and the aqueous phase.

Collectively, these observations suggest that Intralipid solubilizes BPD in an aqueous solution and decreases its aggregation-induced quenching while also decreasing the rate of BPD photobleaching and RMS generation. These trends are further supported by recent studies indicating that Intralipid reduces O12 generation and luminescence lifetime of PpIX and Rose Bengal in aqueous solutions.^45^^,^^46^ The diminished generation of O12 has been attributed to a prolonged photosensitizer triplet state residence time under increased scattering conditions, which subsequently leads to decreased energy transfer efficiency.^52^ Interestingly, a similar decrease in fluorescence intensity (normalized to BPD fluorescence) was observed with HPF in escalating concentrations of Intralipid, upon light exposure.

Although the observed effects of Intralipid on photochemically generated RMS are consistent with the literature, the methodologies employed in this study are not without limitations. A primary concern is the potential lack of specificity of the fluorescence probes used to detect type I and type II RMS. For instance, although HPF is generally considered more sensitive to •OH than O12,^51^ Price et al.^44^ demonstrated that HPF can also react to O12 under PDT conditions. This cross-reactivity complicates the interpretation of the effects of Intralipid on type I ROS generation when using HPF as a probe. In addition, although SOSG and HPF fluorescence signals were normalized to BPD fluorescence within each Intralipid condition, this correction does not fully account for scattering-related effects or potential spectral distortions introduced by Intralipid. As a result, the presented RMS data should be regarded as semi-quantitative and warrant further validation through more direct methods, such as time-resolved O12 luminescence measurements.

### 3.2 Photochemical Generation of Lipid Radicals in Intralipid

We hypothesized that the relative decrease in both SOSG and HPF-detected RMS, along with reduced BPD photobleaching, may at least partially stem from oxidation-sensitive unsaturated lipids in Intralipid reacting with and trapping photochemically generated RMS. To test this, we used C11 BODIPY 581/591—a lipophilic, ratiometric probe containing a polyunsaturated butadienyl side chain that is susceptible to oxidation by lipid radicals.^53^^,^^54^ The probe integrates into lipid droplets, and an increase in the oxidized to total (oxidized + unoxidized) C11 BODIPY fluorescence ratio indicates the presence of photochemically-generated lipid radicals.

**Fig. 2:**
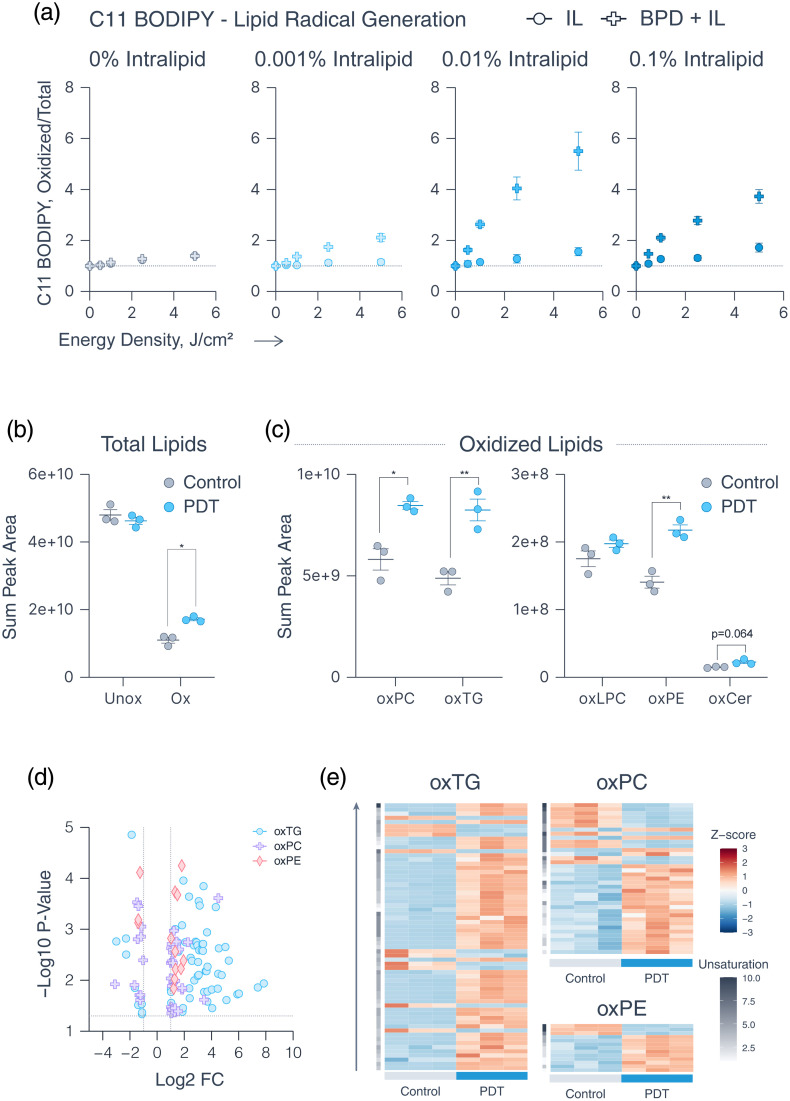
Generation of lipid radicals and oxidized lipids in PDT-exposed Intralipid. (a) Photodynamic generation of lipid radicals in 1  μM BPD solutions containing increasing concentrations of Intralipid under light irradiation. (b) LC-MS analysis of Intralipid showing summed peak areas of unoxidized (Unox) versus oxidized (Ox) lipids, where oxidized lipids contain ─OH and ─OOH groups. (c) Summed peak areas of the five most abundant oxidized lipid classes present in Intralipid. (d) Volcano plot displaying individual oxTG, oxPC, and oxPE species with absolute log2 fold change >1 and −log 10 p-value<0.05. (e) Heatmaps of Z-scores for individual oxTG, oxPC, and oxPE species, arranged in order of increasing carbon chain length (gray arrow) and the total number of double bonds (unsaturation, side bar). Statistics: (a) Each point represents the mean of three independent repeats, each conducted in duplicate; error bars indicate the standard error of the mean. (b), (c) Each point shows the mean of an independent repeat (n=3), each conducted in duplicate; error bars represent the standard error of the mean. Differences between control and PDT groups were assessed using two-tailed, two-sample t-tests (*p<0.05, **p<0.01). Graphs (a)–(d) were created with Graphmatik; heatmaps generated in R.

In a 1  μM BPD solution prepared in PBS, little to no increase in the oxidized/total C11 BODIPY fluorescence ratio was observed, indicating minimal presence of lipid radicals [Fig. 2(a)]. However, all Intralipid-containing solutions showed a significant increase in C11 BODIPY oxidized/total ratio. Notably, the greatest increase in the oxidized/total C11 BODIPY ratio occurred at 0.01% Intralipid (1.56±0.28 at 0% Intralipid, 5.5±0.75 at 0.01% Intralipid at $5\;J/{\mathrm{cm}}^{2}$), whereas a lower ratio was seen at 0.1% Intralipid (1.72±0.17 at 0% Intralipid, 3.7±0.3 at 0.01% Intralipid at $5\;J/{\mathrm{cm}}^{2}$). A decrease in lipid radical generation from 0.01% to 0.1% Intralipid may indicate that an excess of lipids may further suppress the generation of RMS of type I and type II RMS through mechanisms other than lipid radical generation.

### 3.3 PDT-Induced Changes in Intralipid Composition

As a notable generation of lipid radicals was observed in Intralipid solutions following PDT, we hypothesized that PDT induces lipid peroxidation. Using an LC-MS-based lipidomic workflow, we characterized compositional changes in Intralipid after PDT. Among the 1582 lipid species identified via LipidSearch, 589 species containing –OH and –OOH groups were classified as “oxidized,” and 993 as “unoxidized.” Although the summed peak area of unoxidized lipids remained unchanged, a significant increase in oxidized lipids was observed in PDT-exposed Intralipid [$1.1\times 10^{10}\pm 8.7\times 10^{8}$ versus $1.7\times 10^{10}\pm 4.5\times 10^{8}$, Fig. 2(b)]. Of the 16 lipid classes detected, oxidized phosphatidylcholine (oxPC), triacylglycerols (oxTG), lysophosphatidylcholine (oxLPC), phosphatidylethanolamine (oxPE), and ceramides (oxCer) were the five most abundant in the oxidized group [Fig. 2(c), Figs. S9 and S10 in the Supplementary Material]. PDT significantly increased the levels of oxPC ($5.8\times 10^{9}\pm 5.3\times 10^{8}$ versus $8.5\times 10^{9}\pm 1.9\times 10^{8}$), oxTG ($4.9\times 10^{9}\pm 3.3\times 10^{8}$ versus $8.2\times 10^{9}\pm 5.4\times 10^{8}$), and oxPE ($1.4\times 10^{8}\pm 8.78\times 10^{6}$ versus $2.2\times 10^{8}\pm 7.7\times 10^{6}$). Although oxLPC levels remained unchanged, oxCer displayed a strong upward trend ($1.48\times 10^{7}\pm 5.7\times 10^{6}$ versus $2.2\times 10^{7}\pm 2.2\times 10^{6}$).

To further investigate the observed changes, we analyzed individual lipid species contributing to the oxidation profile. We selected oxTG, oxPC, and oxPE species that exhibited an absolute log2 fold change greater than 1 and a p-value<0.05 [Fig. 2(d), Fig. S10 and Table S1 in the Supplementary Material]. Heatmap analysis of Z-scores for oxTG, oxPC, and oxPE species—organized by increasing carbon chain length and degree of unsaturation—revealed distinct patterns of lipid oxidation [Fig. 2(d), Fig. S11 in the Supplementary Material]. Although oxTGs appear to be upregulated regardless of lipid chain length and the total number of double bonds, upregulated oxPCs and oxPEs appear to cluster toward shorter chain lengths and lower unsaturation. Surprisingly, a subset of long-chain, highly unsaturated oxPCs and oxPEs (7+ total double bonds, likely containing two polyunsaturated fatty acyl chains) showed decreased abundance. This likely indicates that highly unsaturated PCs and PEs may already exist in a pre-oxidized state due to storage and are further degraded upon PDT-induced oxidative stress. These findings highlight the broad oxidative capacity of PDT, which not only initiates lipid peroxidation but may also promote degradation of pre-oxidized polyunsaturated lipids across multiple classes and species.

### 3.4 Intralipid Toxicity in the Dark

**Fig. 3:**
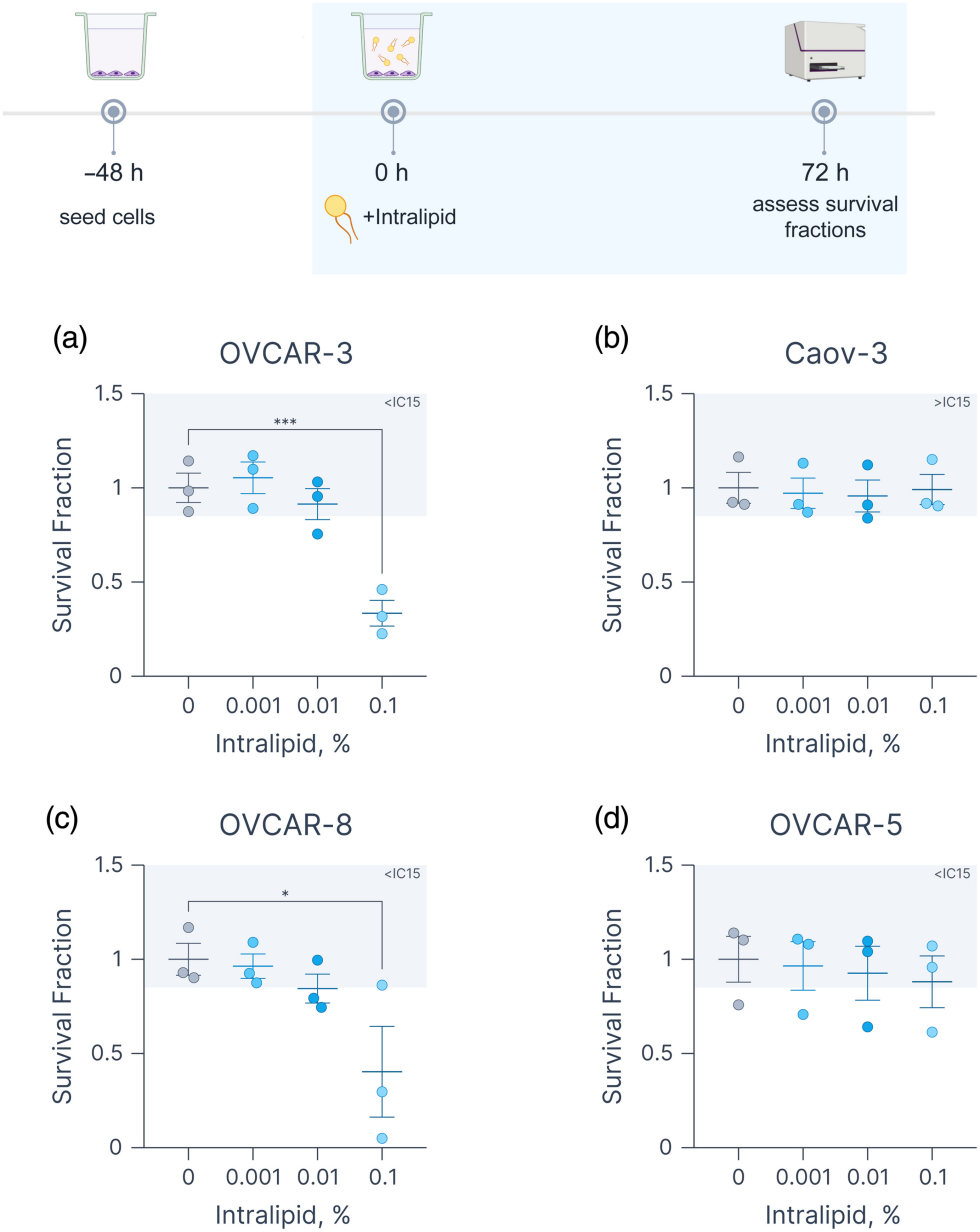
Cytotoxicity of increasing concentrations of Intralipid (0% to 0.1%). Survival fractions of (a) OVCAR-3, (b) Caov-3, (c) OVCAR-8, and (d) OVCAR-5 cells exposed to 0.001% to 0.1% Intralipid. Each point represents an independent experiment (mean of two technical replicates). Statistical differences between groups were evaluated using one-way ANOVA with Dunnett’s correction for multiple comparisons (*p≤0.05, ***p≤0.001), performed in Graphmatik. The timeline schematic was generated using BioRender.

To evaluate the concentration-dependent toxicity of Intralipid in ovarian cancer cell lines, OVCAR-3, Caov-3, OVCAR-8, and OVCAR-5, cells were incubated for 72 h in their respective complete culture media containing 0.001%, 0.01%, or 0.1% Intralipid (Fig. 3). Notably, OVCAR-3 and OVCAR-8 cells exhibited significant reductions in survival fractions at 0.1% Intralipid (0.33±0.07 and 0.42±0.24, respectively). By contrast, OVCAR-5 and Caov-3 cells showed no significant changes in viability. These differential responses underscore the nonbenign biological effects of Intralipid, particularly in ovarian cancer cells that depend on reprogrammed lipid metabolism for survival and proliferation.^55^^,^^56^ It is particularly interesting that both OVCAR-3 and OVCAR-8 cells are metastatic lines derived from patient ascites, characterized by high levels of platinum resistance.^57^^,^^58^ Caov-3 cells are likely to represent primary HGSOC,^57^ whereas the origin of OVCAR-5 cells remains under debate.^18^^,^^59^^,^^60^ Within this context, the increased sensitivity of OVCAR-3 cells to lipid overload, especially concerning unsaturated lipids, compared with Caov-3 cells, aligns with our recent findings indicating their heightened sensitivity to both chemical and photodynamic lipid peroxidation (Overchuk et al.^61^).

### 3.5 Effects of Intralipid on PDT Efficacy

To model the effects of Intralipid on PDT efficacy, we considered two simplified scenarios: short drug-light interval PDT (SDLI-PDT) and conventional PDT. In the SDLI-PDT model, cells were irradiated in the presence of 1  μM BPD in complete culture medium with or without Intralipid (0.01%). Given the brief exposure time (<5  min), minimal internalization of both BPD and Intralipid was assumed, with the primary site of photodamage localized to the plasma membrane. Furthermore, in this case, photodamage to Intralipid was maximized because it was present in solution together with BPD. In the conventional PDT model, cells were first incubated with 0.125  μM BPD for 90 min. This allowed for photosensitizer internalization and redistribution, with mitochondria and endoplasmic reticulum being the primary sites.^62^^,^^63^ The photosensitizer was then removed and replaced with complete culture medium with or without Intralipid, followed by light irradiation. To assess the “dark effects” of PDT-exposed Intralipid in both scenarios, it was either removed immediately after irradiation (“remove”) or left in the medium for 72 h (“leave”).

#### 3.5.1 SDLI-PDT

**Fig. 4:**
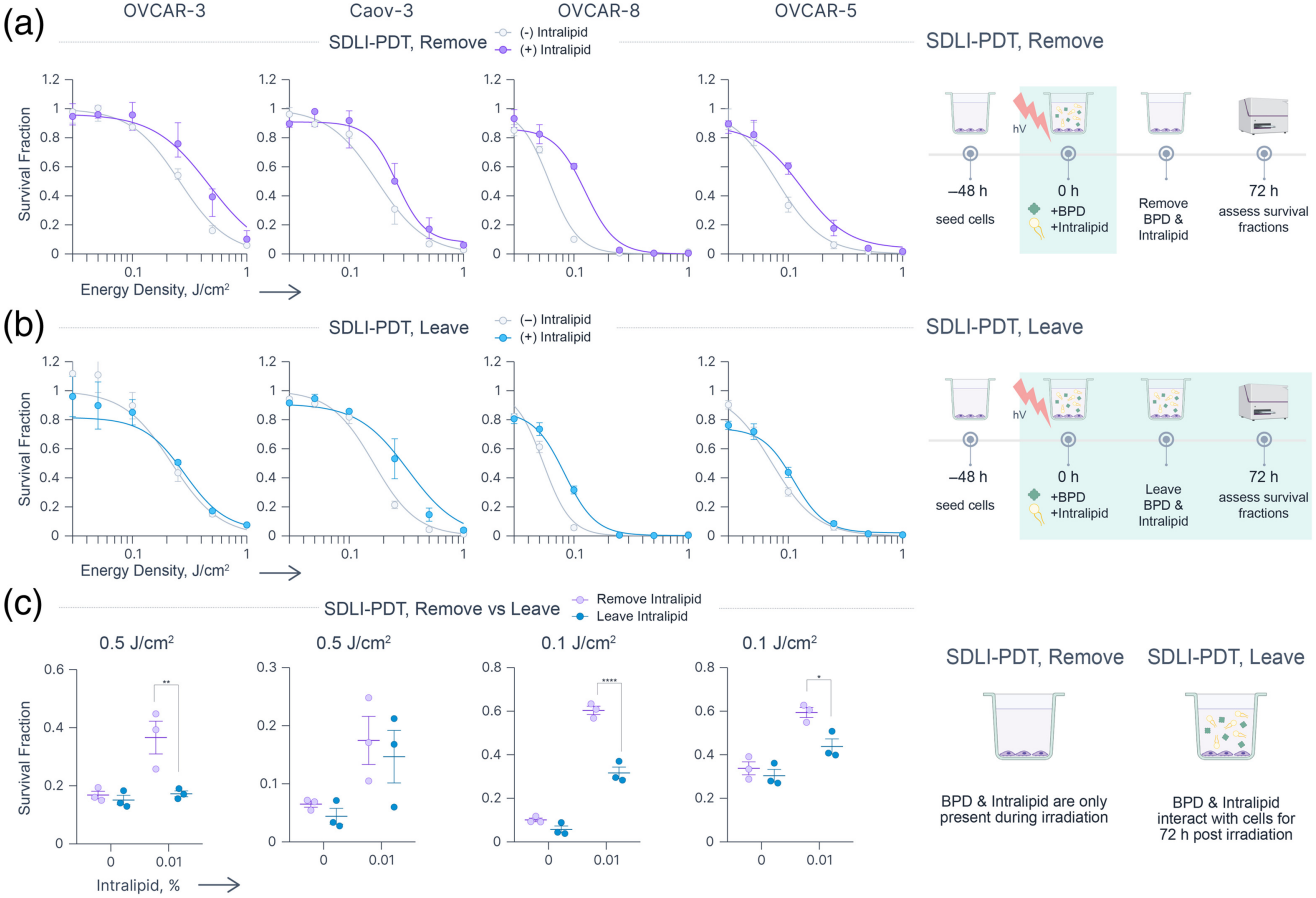
Effects of 0.01% Intralipid on the efficacy of SDLI-PDT in ovarian cancer cell lines. (a) Dose-response curves for OVCAR-3, Caov-3, OVCAR-8 and OVCAR-5 cells treated with SDLI-PDT using 1  μM BPD alone (0%, gray) or with 0.01% Intralipid (purple), where BPD and Intralipid were removed immediately after irradiation. (b) Dose-response curves under the same conditions as in panel (a), but BPD and Intralipid were left in the plates for 72 h post-irradiation. (c) Effect of post-irradiation presence of the BPD-Intralipid mixture at a single energy density across the four cell lines. Statistics: For panels (a) and (b), each point represents the mean of three independent repeats (each with two technical replicates); error bars show the standard error of the mean. Least-squares fit curves are shown (gray, purple, blue), with *R*^2^ > 0.97. For panel (c), each point represents the mean of an independent repeat (each with two technical replicates). Statistical differences between “remove” and “leave” groups were assessed using two-way ANOVA with Sidak’s multiple comparisons correction. All graphs were generated in Graphmatik; statistical analysis was performed using GraphPad Prism. All schematics were generated using BioRender.

Overall, the addition of Intralipid to BPD-containing medium significantly reduced cell killing across all four cell lines during SDLI-PDT. This effect was particularly pronounced in “remove” conditions [Fig. 4(a), Table S2 in the Supplementary Material], where the BPD-Intralipid-containing medium was removed immediately post-PDT and replaced with fresh medium. Given the observed and reported^3^^,^^45^^,^^46^ decrease in RMS generation in Intralipid-containing solutions, the increased post-PDT survival fractions are likely a direct result of this reduced oxidative insult.

Next, we investigated the effects of the light-exposed BPD-Intralipid mixture by allowing it to remain in the wells after PDT for 72 h [“leave” condition, Fig. 4(b), Table S3 in the Supplementary Material]. Interestingly, the resulting dose responses under the “leave” condition more closely resembled those observed without Intralipid. This suggests that any initial reduction in RMS cytotoxicity was compensated for by continued cell exposure to potentially cytotoxic Intralipid oxidation products. Indeed, a direct comparison of SDLI-PDT cytotoxicity between “remove” and “leave” conditions at 0.5 or $0.1\;J/{\mathrm{cm}}^{2}$ demonstrated that the light-exposed BPD-Intralipid mixture had significant cytotoxic effects in three out of the four cell lines tested [Fig. 4(c)]. Given that the cytotoxic properties of thermally- or UV-oxidized lipids are well documented, it is unsurprising that PDT-exposed Intralipid exhibits similar effects.^64^^,^^65^

#### 3.5.2 Conventional PDT

**Fig. 5:**
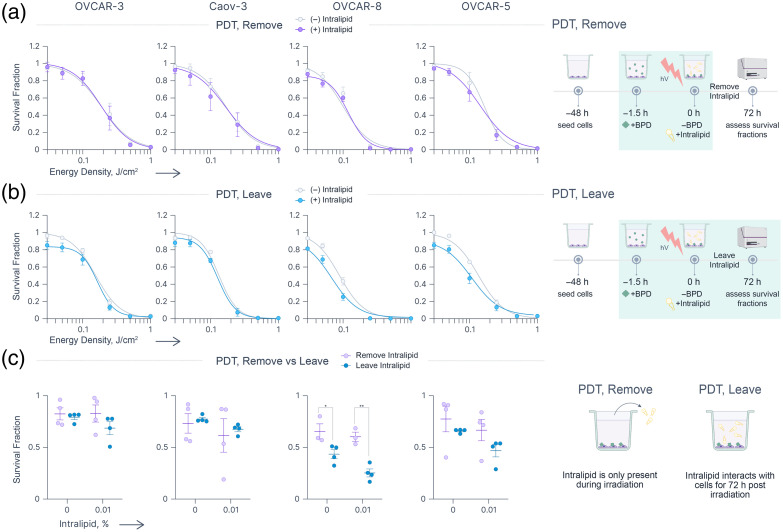
Effects of 0.01% Intralipid on the efficacy of PDT in ovarian cancer cell lines. (a) Dose-response curves for OVCAR-3, Caov-3, OVCAR-8, and OVCAR-5 cells treated with PDT using 0.125  μM BPD alone (0%, gray) or with 0.01% Intralipid (purple), where BPD was incubated with cells for 90 min, and Intralipid was added prior to and removed immediately after irradiation. (b) Dose-response curves under the same conditions as in panel (a) and Intralipid were left in the plates for 72 h post-irradiation. (c) Effect of post-irradiation presence of PDT-exposed Intralipid at a $0.1\;J/{\mathrm{cm}}^{2}$ across the four cell lines. Statistics: For panels (a) and (b), each point represents the mean of four independent repeats; error bars show the standard error of the mean. Least-squares fit curves are shown (gray, purple, blue), with $R^{2}>0.97$. For panel (c), each point represents the mean of an independent replicate (each with two technical replicates). Statistical differences between “remove” and “leave” groups were assessed using two-way ANOVA with Sidak’s multiple comparisons correction. All graphs were generated in Graphmatik; statistical analysis was performed using GraphPad Prism. All schematics were generated using BioRender.

Next, we evaluated the effects of Intralipid on conventional PDT. When Intralipid was added immediately prior to PDT and subsequently removed immediately after (“remove” wells), no significant differences in cell survival were observed, apart from increased variability [Fig. 5(a), Table S4 in the Supplementary Material]. In this scenario, BPD would have already been internalized by the cells, whereas Intralipid was only transiently present extracellularly, thus explaining the lack of interaction. Interestingly, a slight trend toward decreased cell survival was noted in “leave” wells, likely corresponding to minor cytotoxic effects of the Intralipid itself [Fig. 5(b), Table S5 in the Supplementary Material].

Furthermore, although the difference between “remove” and “leave” conditions was nonsignificant for Caov-3 cells, OVCAR-3 and OVCAR-5 cells exhibited a trend toward reduced survival fraction, and OVCAR-8 cells showed a significant reduction in survival fraction in the “leave” condition, regardless of the presence of Intralipid [Fig. 5(c)]. We hypothesize that components within the FBS and cell culture medium that come into contact with even small concentrations of BPD (either remaining in wells post-removal or leaking from cells during irradiation) may exert minor cytotoxic effects similar to PDT-exposed Intralipid. Given the strong sensitivity of OVCAR-8 cells to Intralipid (Fig. 3), it is plausible that they are also susceptible to low concentrations of oxidized lipids originating from FBS.

### 3.6 Cytotoxic Effects of PDT-Exposed Intralipid

**Fig. 6:**
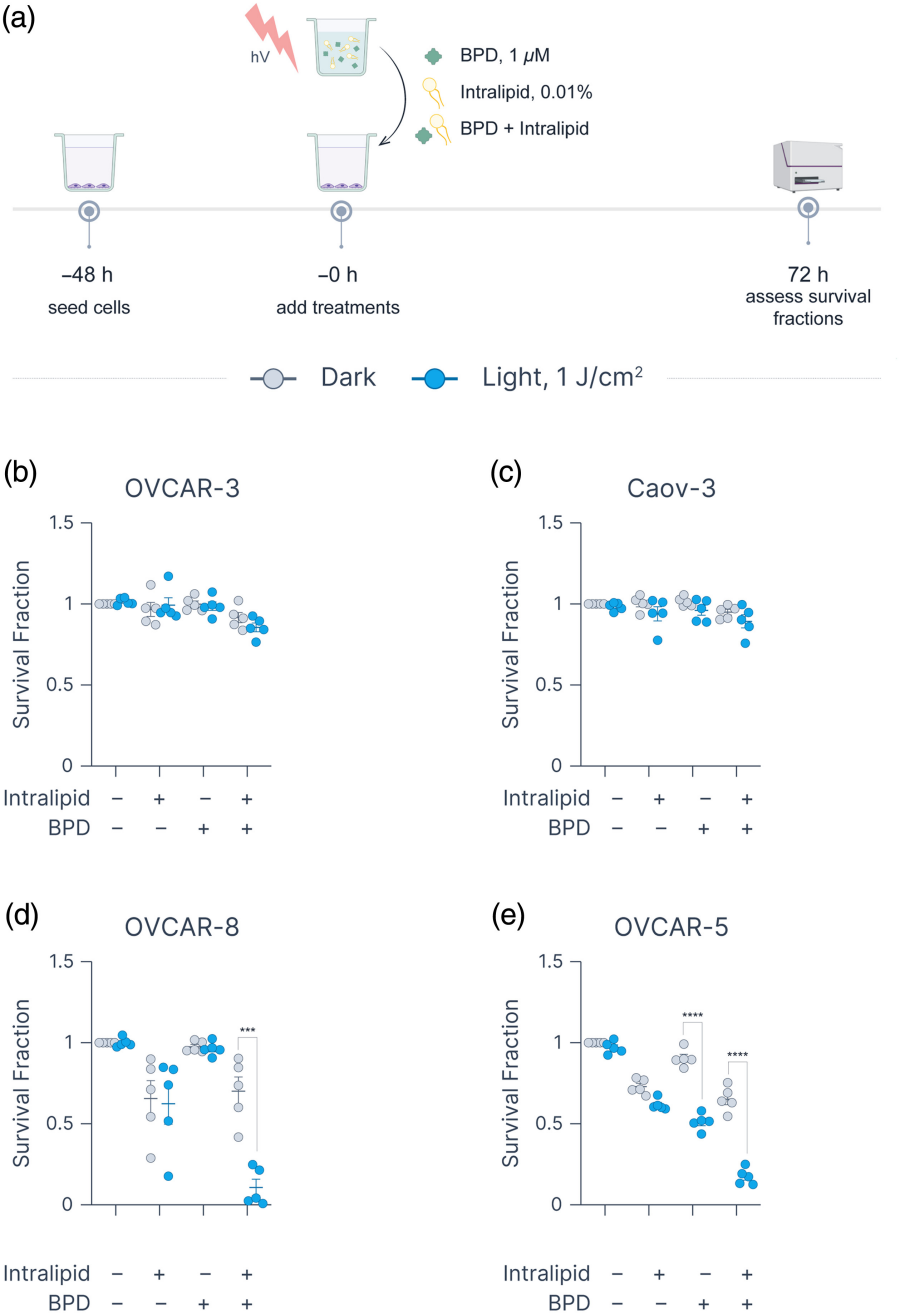
Effects of photoexposed BPD, Intralipid, and BPD–Intralipid mixtures on cell survival in ovarian cancer lines. (a) Experimental workflow schematic (created using BioRender). Survival fractions for (b) OVCAR-3, (c) Caov-3, (d) OVCAR-8, and (e) OVCAR-5 cells. Statistics: Each point represents the mean of an independent experiment, each performed in duplicate; error bars indicate the standard error of the mean. Statistical differences between “dark” and “light, $1\;J/{\mathrm{cm}}^{2}$” conditions within each treatment group were evaluated using a t-test (***p≤0.001, ****p≤0.0001). All graphs were generated using Graphmatik.

To directly assess the cytotoxicity of pre-oxidized Intralipid, we conducted a separate set of experiments in which complete cell culture media containing Intralipid (0.01%), BPD (1  μM), or a BPD–Intralipid mixture were either kept in the dark or exposed to $1\;J/cm^{2}$ of 690 nm light. Immediately after irradiation, these solutions were transferred to plates containing untreated cells and incubated for 72 h. Although OVCAR-3 and Caov-3 cells displayed minimal sensitivity to any condition, both OVCAR-8 and OVCAR-5 cells exhibited pronounced cytotoxic responses (Fig. 6). Specifically, survival fractions significantly decreased upon exposure to photoactivated BPD–Intralipid mixtures (OVCAR-8: 0.70±0.09 in dark versus 0.11±0.05 after light; OVCAR-5: 0.65±0.03 in dark versus 0.17±0.02 after light). These outcomes are consistent with the PDT experiments, where OVCAR-8 cells showed the largest survival differences between “leave” and “remove” conditions, suggesting heightened sensitivity to oxidation products formed in Intralipid. Interestingly, OVCAR-5 cells also responded to light-protected BPD, supporting prior findings of BPD exhibiting light-independent cytotoxicity in certain cell lines.^66^ Further experiments across a wider range of light doses (up to $10\;J/cm^{2}$) revealed that OVCAR-3 and Caov-3 cells remained largely resistant to both BPD and Intralipid, alone or in combination, whereas OVCAR-8 and OVCAR-5 cells were completely eradicated under the same conditions (Fig. S12 in the Supplementary Material). Together, these results highlight a striking variability in cell line responses to BPD, Intralipid, and their oxidation products, emphasizing the need for further investigation into the mechanistic underpinnings of this differential sensitivity.

## 4 Conclusion

Our findings support the emerging view that Intralipid is not merely a passive scattering agent but can also modulate PDT response. When co-administered with BPD, Intralipid reduces RMS generation relative to BPD fluorescence at equivalent Intralipid concentrations. Conversely, Intralipid readily forms lipid radicals upon exposure to light and a photosensitizer, suggesting a possible shift in the balance of PDT-induced cytotoxic mechanisms—from O12-mediated damage to lipid radical- and hydroperoxide-induced cytotoxicity.

Notably, the impact of Intralipid on *in vitro* PDT efficacy is highly dependent on the spatial and temporal relationship between Intralipid and the photosensitizer (Table 1). The most pronounced reduction in PDT efficacy occurs when both Intralipid and BPD are present in the cell culture medium, simulating intraperitoneal co-administration. Interestingly, BPD–Intralipid mixtures exposed to light exhibit dark cytotoxicity, resulting in notable decreases in survival fractions after 72 h of incubation in three out of four cell lines. The increased cytotoxicity of PDT-exposed Intralipid is likely attributable to significant lipid oxidation, as demonstrated by LC-MS analysis, showing widespread oxidation across multiple lipid classes, most notably PC, PE, and TG, following PDT exposure. By contrast, when BPD is predominantly internalized by cells and Intralipid remains extracellular—loosely mimicking intravenous photosensitizer delivery with longer photosensitizer-light intervals—Intralipid exerts minimal effect on PDT outcome.

**Table 1:** Summary of the effects of Intralipid on survival fractions and BPD-PDT efficacy in ovarian cancer cell lines. Arrows indicate changes in survival fractions relative to matched conditions without Intralipid.

Treatment	Dark toxicity	SDLI-PDT		Conventional PDT	
Exposures	Intralipid, 0.1%	Intralipid (0.01%) + BPD (1 μM) + hv, 0 to $1\;J/{\mathrm{cm}}^{2}$		Intralipid (0.01%) + BPD (0.125 μM) + hv, 0 to $1\;J/{\mathrm{cm}}^{2}$	
Intralipid exposure duration	72 h	Remove (<5 min)	Leave (72 h)	Remove (<5 min)	Leave (72 h)
OVCAR-3	↓	↑	No change	No change	No change
Caov-3	No change	↑	↑	No change	No change
OVCAR-8	↓	↑	↑	No change	↓
OVCAR-5	No change	↑	No change	No change	↓

Although this study clearly demonstrates that Intralipid significantly modulates the outcomes of PDT, further *in vivo* validation is essential. The experimental frameworks used here—comparing conventional PDT with SDLI-PDT, and “leave” versus “remove” conditions—were intentionally designed as conceptual endpoints to illustrate the range of possible effects. *In vivo*, however, we anticipate a far more nuanced scenario, likely incorporating elements of both extremes. For example, BPD may distribute both intracellularly and extracellularly, whereas Intralipid oxidation products—initially present—will gradually clear through circulation. Our *in vitro* findings further underscore the importance of administration route and timing for both BPD and Intralipid as these factors will likely influence therapeutic efficacy. Yet, translating these insights *in vivo* poses unique challenges, including differences in tissue light scattering depending on Intralipid presence. Overcoming such challenges may require the development of new Intralipid formulations—for instance, by tuning lipid saturation levels to control susceptibility to oxidation and, in turn, mitigate cytotoxicity.

In summary, this study demonstrates that Intralipid significantly reduces RMS generation in solution, whereas its components undergo extensive PDT-induced lipid oxidation. These two processes exert opposing effects on PDT response: the reduction of RMS generation may protect cells from immediate PDT-induced cytotoxicity, whereas the formation of oxidized lipids may contribute to delayed cell death. Further research is warranted to elucidate the broader cytotoxic effects^64^^,^^65^^,^^67^^,^^68^ of Intralipid-derived oxidation products generated during PDT. Moreover, given recent reports suggesting that certain oxidized lipids may exert immunogenic effects,^69^^–^^71^ the potential systemic immune impact of both naïve and photochemically oxidized Intralipid warrants further investigation.

## 5 Supplementary Material

10.1117/1.JBO.30.S3. S34116.s01

## Data Availability

All supporting data and relevant analyses will be made available at UNC Dataverse, a public data repository service for the University of North Carolina at Chapel Hill (UNC) research community and can be accessed using the following link: https://dataverse.unc.edu/dataverse/Overchuk_JBO_2025.

## References

[1] Raman M. et al., “Parenteral nutrition and lipids,” Nutrients 9(4), 388 (2017). 10.3390/nu9040388 28420095 PMC5409727

[2] Jeppesen P. B. Hoy C. E. Mortensen P. B., “Essential fatty acid deficiency in patients receiving home parenteral nutrition,” Amer. J. Clin. Nutr. 68(1), 126–133 (1998). 10.1093/ajcn/68.1.126 9665106

[3] Durantini A. M. et al., “Photooxidative vulnerability to intralipid in photodynamic therapy,” in Photochemistry: Volume 48, Protti S. Raviola C., Eds., The Royal Society of Chemistry (2020).

[4] Driver I. et al., “The optical properties of aqueous suspensions of Intralipid, a fat emulsion,” Phys. Med. Biol. 34(12), 1927 (1989). PHMBA7 0031-9155 10.1088/0031-9155/34/12/015

[5] Flock S. T. et al., “Optical properties of intralipid: a phantom medium for light propagation studies,” Lasers Surg. Med. 12(5), 510–519 (1992). LSMEDI 0196-8092 10.1002/lsm.1900120510 1406004

[6] Agostinis P. et al., “Photodynamic therapy of cancer: an update,” CA Cancer J. Clin. 61(4), 250–281 (2011). CAMCAM 0007-9235 10.3322/caac.20114 21617154 PMC3209659

[7] Wilson B. C. Patterson M. S., “The physics, biophysics and technology of photodynamic therapy,” Phys. Med. Biol. 53(9), R61–R109 (2008). PHMBA7 0031-9155 10.1088/0031-9155/53/9/R01 18401068

[8] Lilge L. et al., “Light dosimetry for intraperitoneal photodynamic therapy in a murine xenograft model of human epithelial ovarian carcinoma,” Photochem. Photobiol. 68(3), 281–288 (1998). PHCBAP 0031-8655 10.1111/j.1751-1097.1998.tb09682.x 9747583

[9] Cengel K. A. Glatstein E. Hahn S. M., “Intraperitoneal photodynamic therapy,” Cancer Treat. Res 134, 493–514 (2007). CTRREP 0927-3042 10.1007/978-0-387-48993-3_34 17633077

[10] Siegel R. L. et al., “Cancer statistics, 2023,” CA Cancer J. Clin. 73(1), 17–48 (2023). CAMCAM 0007-9235 10.3322/caac.21763 36633525

[r11] St Laurent J. Liu J. F., “Treatment approaches for platinum-resistant ovarian cancer,” J. Clin. Oncol. 42(2), 127–133 (2024). JCONDN 0732-183X 10.1200/JCO.23.01771 37910841

[r12] Lheureux S. et al., “Epithelial ovarian cancer,” Lancet 393(10177), 1240–1253 (2019). LANCAO 0140-6736 10.1016/S0140-6736(18)32552-2 30910306

[13] Penn C. A. Alvarez R. D., “Current issues in the management of patients with newly diagnosed advanced-stage high-grade serous carcinoma of the ovary,” JCO Oncol. Pract. 19(3), 116–122 (2023). 10.1200/OP.22.00461 36603168

[14] Lee Y. et al., “A candidate precursor to serous carcinoma that originates in the distal fallopian tube,” J. Pathol. 211(1), 26–35 (2007). 10.1002/path.2091 17117391

[r15] Bowtell D. D. et al., “Rethinking ovarian cancer II: reducing mortality from high-grade serous ovarian cancer,” Nat. Rev. Cancer 15(11), 668–679 (2015). NRCAC4 1474-175X 10.1038/nrc4019 26493647 PMC4892184

[16] Bhamidipati D. et al., “PARP inhibitors: enhancing efficacy through rational combinations,” Br. J. Cancer 129(6), 904–916 (2023). BJCAAI 0007-0920 10.1038/s41416-023-02326-7 37430137 PMC10491787

[17] Azais H. et al., “Dealing with microscopic peritoneal metastases of epithelial ovarian cancer. A surgical challenge,” Surg. Oncol. 26(1), 46–52 (2017). SUOCEC 0960-7404 10.1016/j.suronc.2017.01.001 28317584

[18] Molpus K. L. et al., “Intraperitoneal photodynamic therapy of human epithelial ovarian carcinomatosis in a xenograft murine model,” Cancer Res. 56(5), 1075–1082 (1996). CNREA8 0008-5472 8640764

[r19] Molpus K. L. et al., “Intraperitoneal photoimmunotherapy of ovarian carcinoma xenografts in nude mice using charged photoimmunoconjugates,” Gynecol. Oncol. 76(3), 397–404 (2000). GYNOA3 10.1006/gyno.1999.5705 10684717

[r20] del Carmen M. G. et al., “Synergism of epidermal growth factor receptor-targeted immunotherapy with photodynamic treatment of ovarian cancer in vivo,” J. Natl. Cancer Inst. 97(20), 1516–1524 (2005). JNCIEQ 10.1093/jnci/dji314 16234565

[r21] Spring B. Q. et al., “A photoactivable multi-inhibitor nanoliposome for tumour control and simultaneous inhibition of treatment escape pathways,” Nat. Nanotechnol. 11(4), 378–387 (2016). NNAABX 1748-3387 10.1038/nnano.2015.311 26780659 PMC4821671

[22] Liang B. J. et al., “Fluorescence-guided photoimmunotherapy using targeted nanotechnology and ML7710 to manage peritoneal carcinomatosis,” Sci. Adv. 9(36), eadi3441 (2023). STAMCV 1468-6996 10.1126/sciadv.adi3441 37672582 PMC10482332

[23] Rickard B. P. et al., “Photochemical targeting of mitochondria to overcome chemoresistance in ovarian cancer (dagger),” Photochem. Photobiol. 99(2), 448–468 (2023). PHCBAP 0031-8655 10.1111/php.13723 36117466 PMC10043796

[r24] Overchuk M. et al., “Overcoming the effects of fluid shear stress in ovarian cancer cell lines: doxorubicin alone or photodynamic priming to target platinum resistance,” Photochem. Photobiol. 100(6), 1676–1693 (2024). PHCBAP 0031-8655 10.1111/php.13967 38849970 PMC11568959

[r25] Ruhi M. K. et al., “PpIX-enabled fluorescence-based detection and photodynamic priming of platinum-resistant ovarian cancer cells under fluid shear stress,” Photochem. Photobiol. 100(6), 1603–1621 (2024). PHCBAP 0031-8655 10.1111/php.14014 39189505

[r26] Zuluaga M. F. Lange N., “Combination of photodynamic therapy with anti-cancer agents,” Curr. Med. Chem. 15(17), 1655–1673 (2008). CMCHE7 0929-8673 10.2174/092986708784872401 18673216

[r27] Nath S. et al., “Flow-induced shear stress confers resistance to carboplatin in an adherent three-dimensional model for ovarian cancer: a role for EGFR-targeted photoimmunotherapy informed by physical stress,” J. Clin. Med. 9(4), 924 (2020). 10.3390/jcm9040924 32231055 PMC7230263

[r28] Cramer G. et al., “Preclinical evaluation of cetuximab and benzoporphyrin derivative-mediated intraperitoneal photodynamic therapy in a canine model,” Photochem. Photobiol. 96(3), 684–691 (2020). PHCBAP 0031-8655 10.1111/php.13247 32119123

[29] Obaid G. et al., “Engineering photodynamics for treatment, priming and imaging,” Nat. Rev. Bioeng. 2, 752–769 (2024). 10.1038/s44222-024-00196-z 39927170 PMC11801064

[30] Azais H. Mordon S. Collinet P., “[Intraperitoneal photodynamic therapy for peritoneal metastasis of epithelial ovarian cancer. Limits and future prospects],” Gynecol. Obstet. Fertil. Senol. 45(4), 249–256 (2017). 10.1016/j.gofs.2017.02.005 28373041

[31] Hahn S. M. et al., “Photofrin uptake in the tumor and normal tissues of patients receiving intraperitoneal photodynamic therapy,” Clin. Cancer Res. 12(18), 5464–5470 (2006). 10.1158/1078-0432.CCR-06-0953 17000681

[32] Lilge L. et al., Light Delivery and Dosimetry for Photodynamic Therapy in an Ovarian-Cancer Mouse Model, SPIE Press, Bellingham, Washington (1994).

[33] Sindelar W. F. et al., “Technique of photodynamic therapy for disseminated intraperitoneal malignant neoplasms. Phase I study,” Arch. Surg. 126(3), 318–324 (1991). 10.1001/archsurg.1991.01410270062011 1998474

[r34] DeLaney T. F. et al., “Phase I study of debulking surgery and photodynamic therapy for disseminated intraperitoneal tumors,” Int. J. Radiat. Oncol. Biol. Phys. 25(3), 445–457 (1993). IOBPD3 0360-3016 10.1016/0360-3016(93)90066-5 8436523

[r35] Bauer T. W. et al., “Preliminary report of photodynamic therapy for intraperitoneal sarcomatosis,” Ann. Surg. Oncol. 8(3), 254–259 (2001). 10.1007/s10434-001-0254-7 11314943

[r36] Hendren S. K. et al., “Phase II trial of debulking surgery and photodynamic therapy for disseminated intraperitoneal tumors,” Ann. Surg. Oncol. 8(1), 65–71 (2001). 10.1007/s10434-001-0065-x 11206227

[37] Hahn S. M. et al., “A phase II trial of intraperitoneal photodynamic therapy for patients with peritoneal carcinomatosis and sarcomatosis,” Clin. Cancer Res. 12(8), 2517–2525 (2006). 10.1158/1078-0432.CCR-05-1625 16638861

[38] Pass H. I. Donington J. S., “Use of photodynamic therapy for the management of pleural malignancies,” Semin. Surg. Oncol. 11(5), 360–367 (1995). SSONEV 1098-2388 10.1002/ssu.2980110506 7569558

[r39] Sun H. et al., “Clinical PDT dose dosimetry for pleural Photofrin-mediated photodynamic therapy,” J. Biomed. Opt. 29(1), 018001 (2024). JBOPFO 1083-3668 10.1117/1.JBO.29.1.018001 38223299 PMC10787190

[40] Zhu T. C. et al., “Real-time PDT dose dosimetry for pleural photodynamic therapy,” Proc. SPIE 11940, 1194002 (2022). 10.1117/12.2612188 PMC9104001 35573026

[41] Rahman K. M. M. et al., “Photodynamic therapy for bladder cancers, a focused review (dagger),” Photochem. Photobiol. 99(2), 420–436 (2023). PHCBAP 0031-8655 10.1111/php.13726 36138552 PMC10421568

[42] Manyak M. J. Ogan K., “Photodynamic therapy for refractory superficial bladder cancer: long-term clinical outcomes of single treatment using intravesical diffusion medium,” J. Endourol. 17(8), 633–639 (2003). 10.1089/089277903322518644 14622483

[43] Muller P. J. Wilson B. C., “Photodynamic therapy of brain tumors—a work in progress,” Lasers Surg. Med. 38(5), 384–389 (2006). LSMEDI 0196-8092 10.1002/lsm.20338 16788926

[44] Price M. et al., “Monitoring singlet oxygen and hydroxyl radical formation with fluorescent probes during photodynamic therapy,” Photochem. Photobiol. 85(5), 1177–1181 (2009). PHCBAP 0031-8655 10.1111/j.1751-1097.2009.00555.x 19508643 PMC2745507

[r45] Vikas V. et al., Impact of Scattering Medium on Singlet Oxygen Luminescence Generation in Photodynamic Therapy, SPIE Press, Bellingham, Washington (2025).

[46] Vikas V. et al., “Analysis of singlet oxygen luminescence generated by protoporphyrin IX,” Antioxidants 14(2), 176 (2025). 10.3390/antiox14020176 40002363 PMC11851838

[47] Baptista M. S. et al., “Type I and type II photosensitized oxidation reactions: guidelines and mechanistic pathways,” Photochem. Photobiol. 93(4), 912–919 (2017). PHCBAP 0031-8655 10.1111/php.12716 28084040 PMC5500392

[48] Girotti A. W., “Mechanisms of lipid peroxidation,” J. Free Radic. Biol. Med. 1(2), 87–95 (1985). JFRMEN 0748-5514 10.1016/0748-5514(85)90011-X 3915303

[r49] Girotti A. W., “Photodynamic lipid peroxidation in biological systems,” Photochem. Photobiol. 51(4), 497–509 (1990). PHCBAP 0031-8655 10.1111/j.1751-1097.1990.tb01744.x 2188273

[50] Girotti A. W., “Photosensitized oxidation of membrane lipids: reaction pathways, cytotoxic effects, and cytoprotective mechanisms,” J. Photochem. Photobiol. B 63(1-3), 103–113 (2001). JPPBEG 1011-1344 10.1016/S1011-1344(01)00207-X 11684457

[51] Setsukinai K. et al., “Development of novel fluorescence probes that can reliably detect reactive oxygen species and distinguish specific species,” J. Biol. Chem. 278(5), 3170–3175 (2003). JBCHA3 0021-9258 10.1074/jbc.M209264200 12419811

[52] Hussain M. et al., “Triplet-triplet annihilation photon up-conversion: accessing triplet excited states with minimum energy loss,” J. Photochem. Photobiol. C: Photochem. Rev. 56, 100618 (2023).1389-5567 10.1016/j.jphotochemrev.2023.100618

[53] Drummen G. P. et al., “C11-BODIPY(581/591), an oxidation-sensitive fluorescent lipid peroxidation probe: (micro)spectroscopic characterization and validation of methodology,” Free Radic. Biol. Med. 33(4), 473–490 (2002). FRBMEH 0891-5849 10.1016/S0891-5849(02)00848-1 12160930

[54] Pap E. H. et al., “Ratio-fluorescence microscopy of lipid oxidation in living cells using C11-BODIPY(581/591),” FEBS Lett. 453(3), 278–282 (1999). FEBLAL 0014-5793 10.1016/S0014-5793(99)00696-1 10405160

[55] Zhao G. Cardenas H. Matei D., “Ovarian cancer—why lipids matter,” Cancers 11(12), 1870 (2019). 10.3390/cancers11121870 31769430 PMC6966536

[56] Zhao G. et al., “Ovarian cancer cell fate regulation by the dynamics between saturated and unsaturated fatty acids,” Proc. Natl. Acad. Sci. U. S. A. 119(41), e2203480119 (2022). 10.1073/pnas.2203480119 36197994 PMC9564215

[57] Domcke S. et al., “Evaluating cell lines as tumour models by comparison of genomic profiles,” Nat. Commun. 4, 2126 (2013). NCAOBW 2041-1723 10.1038/ncomms3126 23839242 PMC3715866

[58] Mitra A. K. et al., “In vivo tumor growth of high-grade serous ovarian cancer cell lines,” Gynecol. Oncol. 138(2), 372–377 (2015). GYNOA3 10.1016/j.ygyno.2015.05.040 26050922 PMC4528621

[59] Blayney J. K. et al., “Prior knowledge transfer across transcriptional data sets and technologies using compositional statistics yields new mislabelled ovarian cell line,” Nucleic Acids Res. 44(17), e137 (2016). NARHAD 0305-1048 10.1093/nar/gkw578 27353327 PMC5041471

[60] Renaud M. C. Plante M. Roy M., “Metastatic gastrointestinal tract cancer presenting as ovarian carcinoma,” J. Obstet. Gynaecol. Canada 25(10), 819–824 (2003). 10.1016/S1701-2163(16)30671-5 14532949

[61] Overchuk M. et al., “Photodynamic therapy simultaneously induces ferroptosis- and apoptosis-like lipid signatures in ovarian cancer cells,” Cell Death Dis. (2025). 10.1038/s41419-025-08189-5 PMC12722221 41372113

[62] Rizvi I. et al., “A combination of Visudyne and a lipid-anchored liposomal formulation of benzoporphyrin derivative enhances photodynamic therapy efficacy in a 3D model for ovarian cancer,” Photochem. Photobiol. 95(1), 419–429 (2019). PHCBAP 0031-8655 10.1111/php.13066 30499113 PMC7473467

[63] Kessel D., “Subcellular targeting as a determinant of the efficacy of photodynamic therapy,” Photochem. Photobiol. 93(2), 609–612 (2017). PHCBAP 0031-8655 10.1111/php.12692 27935055 PMC5352468

[64] Alghazeer R. Gao H. Howell N. K., “Cytotoxicity of oxidised lipids in cultured colonal human intestinal cancer cells (CaCo-2 cells),” Toxicol. Lett. 180(3), 202–211 (2008). TOLED5 0378-4274 10.1016/j.toxlet.2008.06.859 18625293

[65] Esterbauer H., “Cytotoxicity and genotoxicity of lipid-oxidation products,” Amer. J. Clin. Nutr. 57(5 Suppl), 779S–786S; discussion 785S-786S (1993). 10.1093/ajcn/57.5.779S 8475896

[66] Baglo Y. et al., “Harnessing the potential synergistic interplay between photosensitizer dark toxicity and chemotherapy,” Photochem. Photobiol. 96(3), 636–645 (2020). PHCBAP 0031-8655 10.1111/php.13196 31856423 PMC7717644

[67] Girotti A. W., “Translocation as a means of disseminating lipid hydroperoxide-induced oxidative damage and effector action,” Free Radic. Biol. Med. 44(6), 956–968 (2008). FRBMEH 0891-5849 10.1016/j.freeradbiomed.2007.12.004 18206663 PMC2361152

[68] Girotti A. W. Korytowski W., “Intermembrane translocation of photodynamically generated lipid hydroperoxides: broadcasting of redox damage,” Photochem. Photobiol. 98(3), 591–597 (2022). PHCBAP 0031-8655 10.1111/php.13537 34633674 PMC8995396

[69] Tyurina Y. Y. et al., “Redox (phospho)lipidomics of signaling in inflammation and programmed cell death,” J. Leukoc. Biol. 106(1), 57–81 (2019). JLBIE7 0741-5400 10.1002/JLB.3MIR0119-004RR 31071242 PMC6626990

[r70] Zhivaki D. Kagan J. C., “Innate immune detection of lipid oxidation as a threat assessment strategy,” Nat. Rev. Immunol. 22(5), 322–330 (2022). NRIABX 1474-1733 10.1038/s41577-021-00618-8 34548649 PMC8454293

[71] Zanoni I. et al., “By capturing inflammatory lipids released from dying cells, the receptor CD14 induces inflammasome-dependent phagocyte hyperactivation,” Immunity 47(4), 697–709.e3 (2017). IUNIEH 1074-7613 10.1016/j.immuni.2017.09.010 29045901 PMC5747599

