# Supplementary material for: Beyond light scattering: the effects of intralipid on benzoporphyrin derivative-sensitized photodynamic treatment in ovarian cancer cells
Source: J Biomed Opt. 2025 Dec 22;30(Suppl 3):S34116. doi: 10.1117/1.JBO.30.S3.S34116 (PMC12770859; doi:10.1117/1.JBO.30.S3.S34116)
Supplement: Supplementary file 1 [file JBO_030_S34116_SD001.pdf]

Supplementary Information

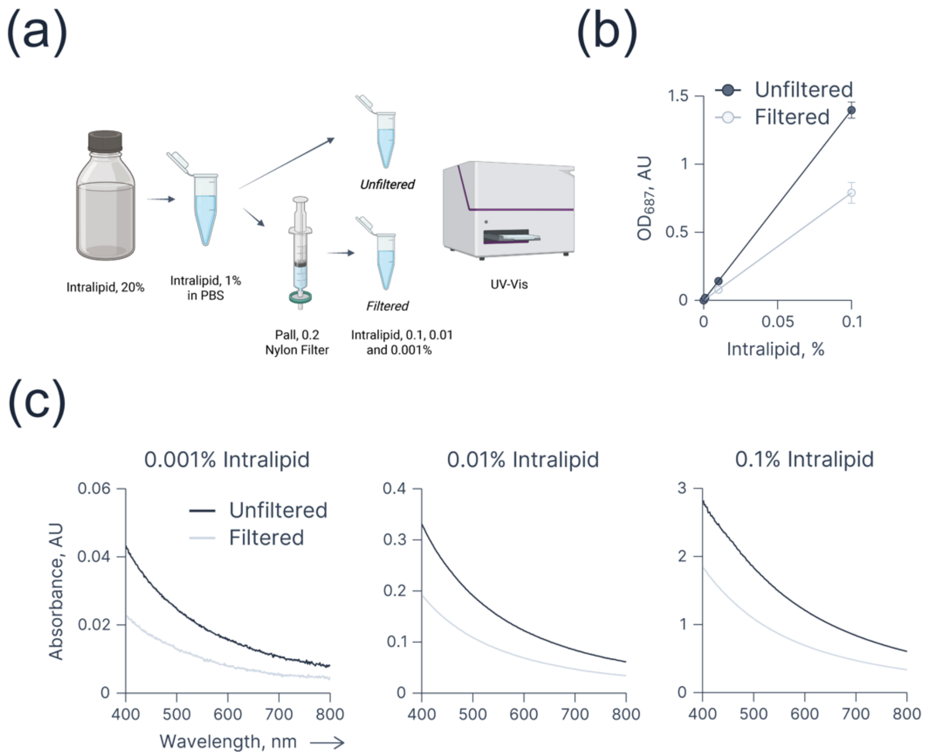

Fig. S1 Effects of Intralipid filtration on optical properties. **(a)** Workflow schematic created in BioRender. Overchuk, M. (2025) https://BioRender.com/srydkvj **(b)** Optical density (OD) at 687 nm and **(c)** UV-Vis absorbance spectra and of unfiltered and filtered 0.1, 0.01 and 0.001% (v/v) Intralipid solutions in PBS. Statistics: Data represent mean values from three independent experiments, each performed in duplicate. R-squared values for panel (b) >0.99.

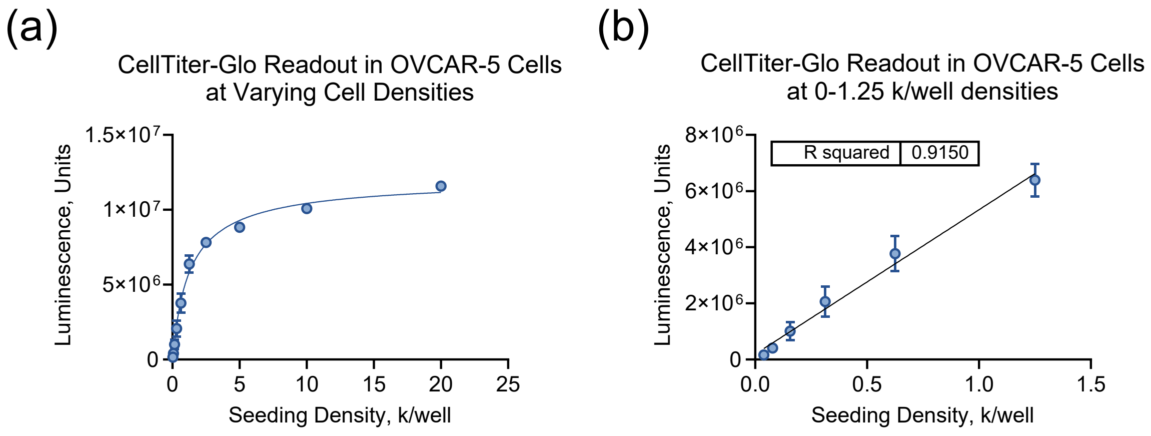

Fig. S2 Determination of the dynamic range for CellTiter-Glo assay for OVCAR-5 cells. **(a)** CellTiter-Glo luminescence signal at 0-20 x 10^4 cells/well and **(b)** 0-1.25 x 10^3 cells/well.

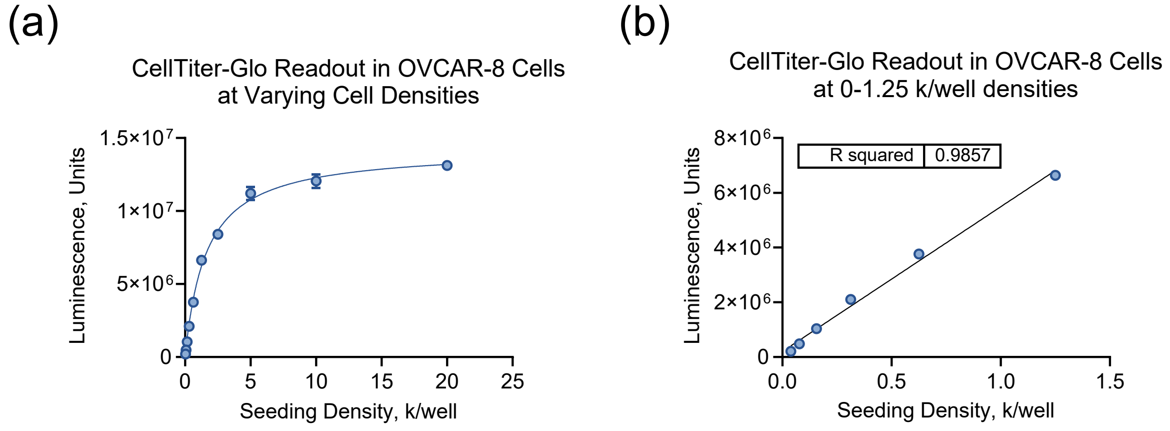

Fig. S3 Determination of the dynamic range for CellTiter-Glo assay for OVCAR-8 cells. **(a)** CellTiter-Glo luminescence signal at 0-2 x 10^4 cells/well and **(b)** 0-1.25 x 10^3 cells/well.

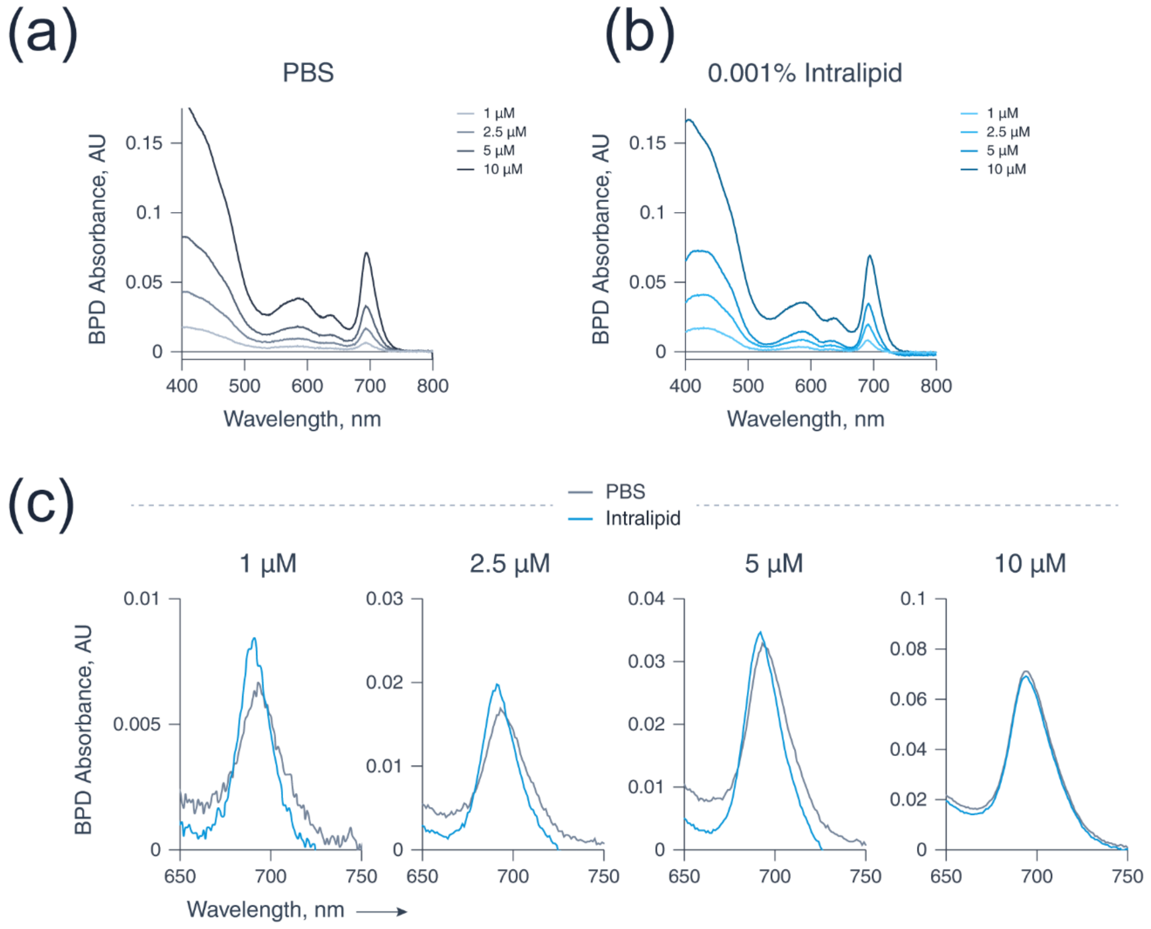

Fig. S4. Effects of Intralipid on BPD absorbance spectrum.

(a) Full BPD absorbance spectrum in PBS and **(b)** in 0.001% Intralipid. **(c)** Q_y peak of the BPD absorbance spectrum (1–10 µM) in PBS and 0.001% Intralipid. Statistics: Data represent mean values from three independent experiments, each performed in duplicate.

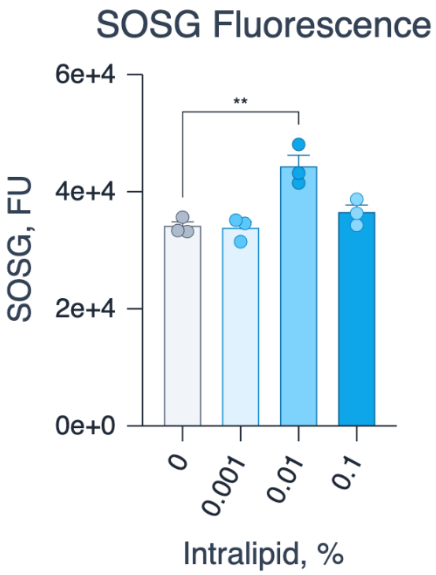

Fig. S5 Fluorescence of Singlet Oxygen Sensor Green (SOSG) in PBS containing 1 µM BPD with or without Intralipid (0–0.1%). Each data point represents the mean of an independent experiment, performed in duplicate. Error bars represent the standard error of the mean.

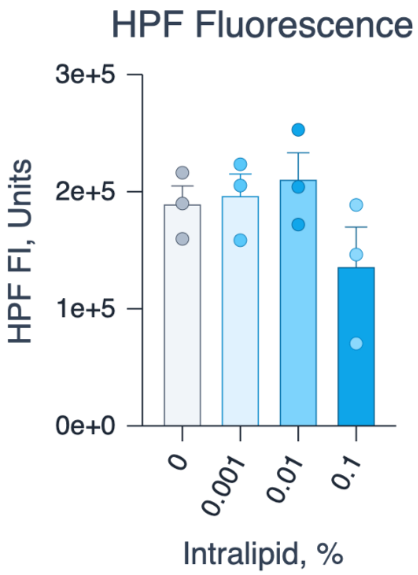

Fig. S6 Fluorescence of hydroxyphenyl fluorescein (HPF) in PBS containing 1 µM BPD with or without Intralipid (0–0.1%). Each data point represents the mean of an independent experiment, performed in duplicate. Error bars represent the standard error of the mean.

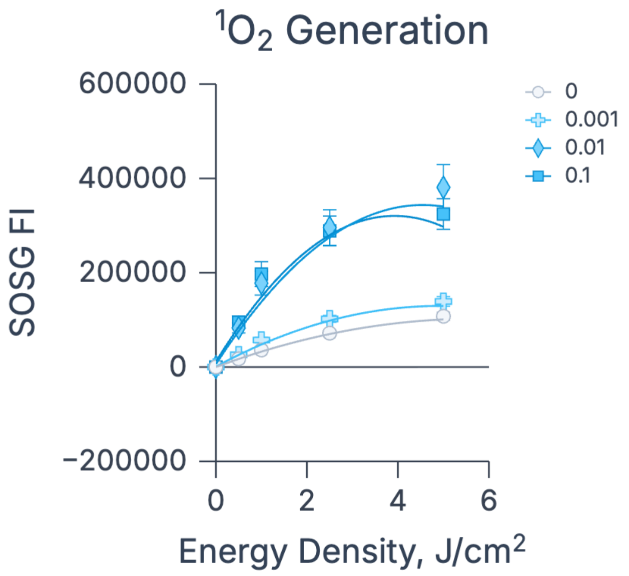

Fig. S7 Fluorescence of Singlet Oxygen Sensor Green in PBS containing 1 µM BPD with or without Intralipid (0–0.1%) upon exposure to 690 nm light (0–5 J/cm²). Each data point represents the mean of three independent experiments, each performed in duplicate. Error bars indicate the standard error of the mean. Connecting lines show a polynomial regression fit ($R^2 > 0.99$).

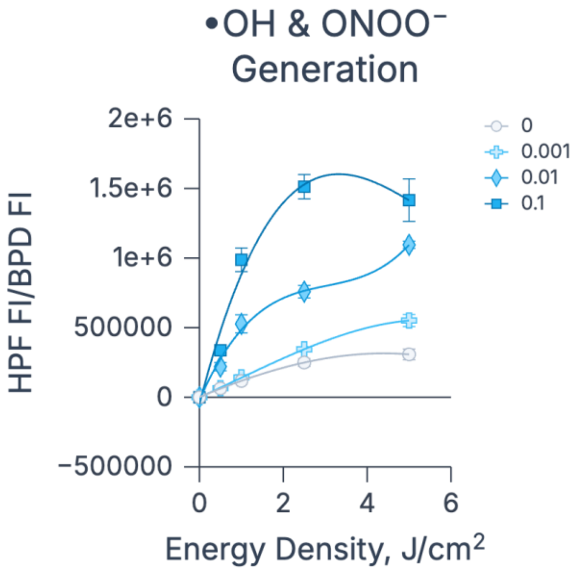

Fig. S8 Fluorescence of Hydroxyphenyl fluorescein (HPF) in PBS containing 1 µM BPD with or without Intralipid (0–0.1%) upon exposure to 690 nm light (0–5 J/cm²). Each data point represents the mean of three independent

experiments, each performed in duplicate. Error bars indicate the standard error of the mean. Connecting lines show a polynomial regression fit ($R^2 > 0.99$).

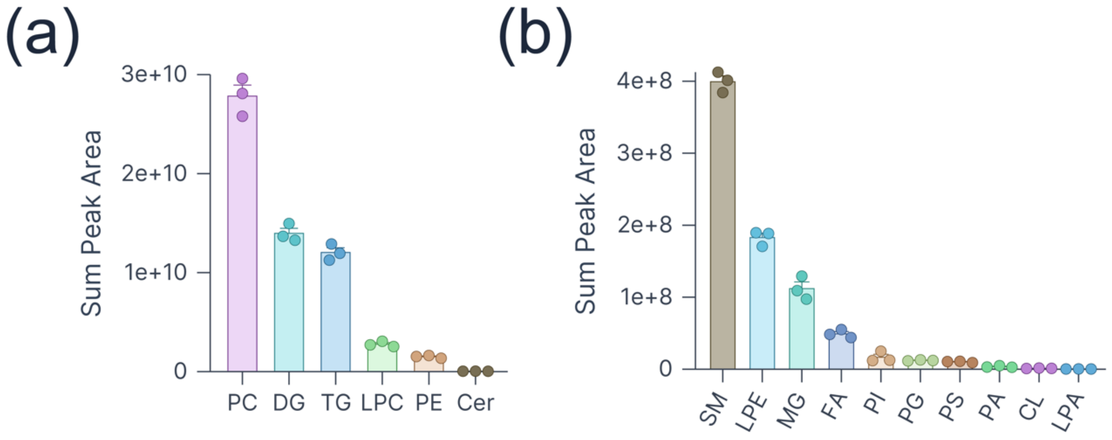

Fig. S9 (a) High-abundance lipid classes, including phosphatidylcholine (PC), diacylglycerol (DG), triacylglycerol (TG), lysophosphatidylcholine (LPC), and phosphatidylethanolamine (PE). **(b)** Low-abundance lipid classes, including sphingomyelin (SM), lysophosphatidylethanolamine (LPE), monoglyceride (MG), free fatty acids (FA), ceramide (Cer), phosphatidylinositol (PI), phosphatidylglycerol (PG), phosphatidylserine (PS), phosphatidic acid (PA), cardiolipin (CL), and lysophosphatidic acid (LPA). Each dot represents an independent experiment, error bars represent the standard error of the mean.

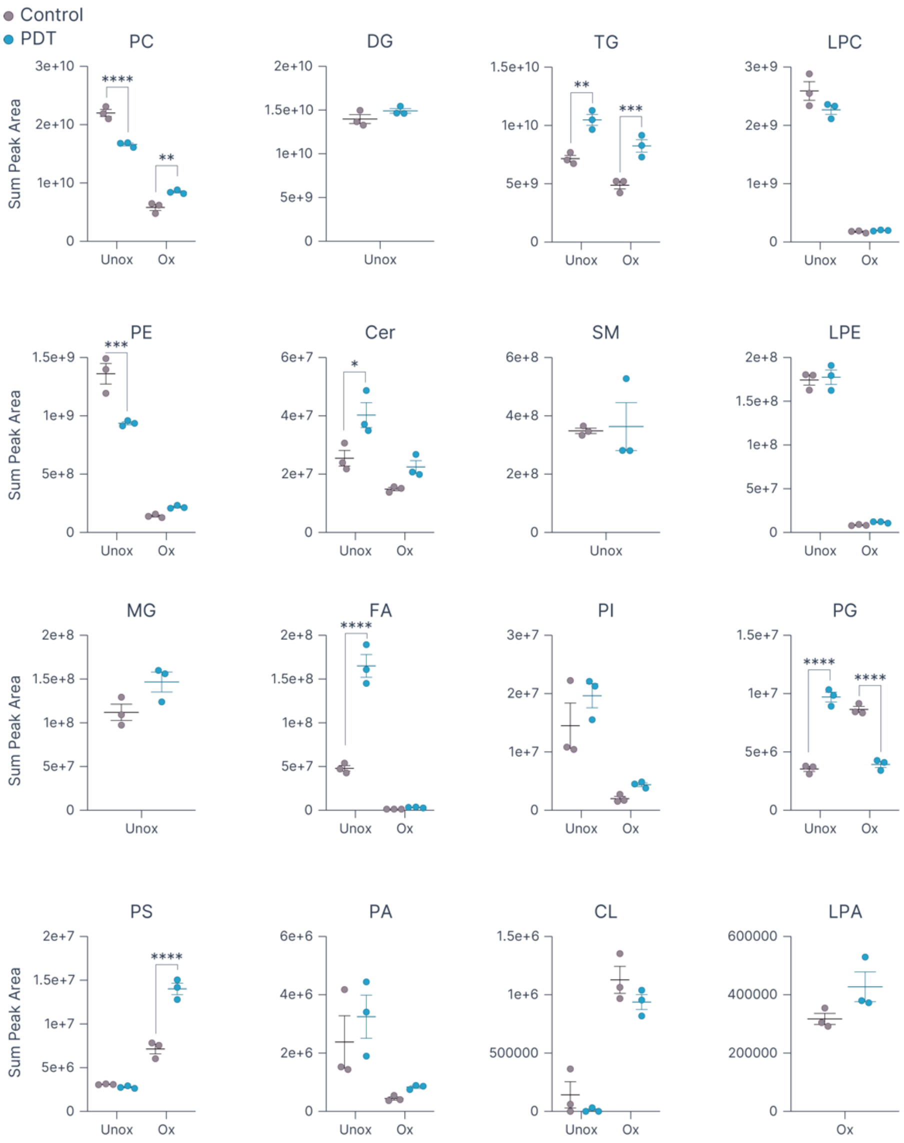

Fig. S10 PDT-induced changes in the summed peak areas of unoxidized (Unox) and oxidized (Ox; containing –OH or –OOH groups) lipids across all classes. Each dot represents an independent experiment; error bars indicate the standard error of the mean. Statistical differences between control and PDT groups were assessed using two-way ANOVA with Šídák's correction for multiple comparisons (GraphPad Prism, Version 10.4.0).

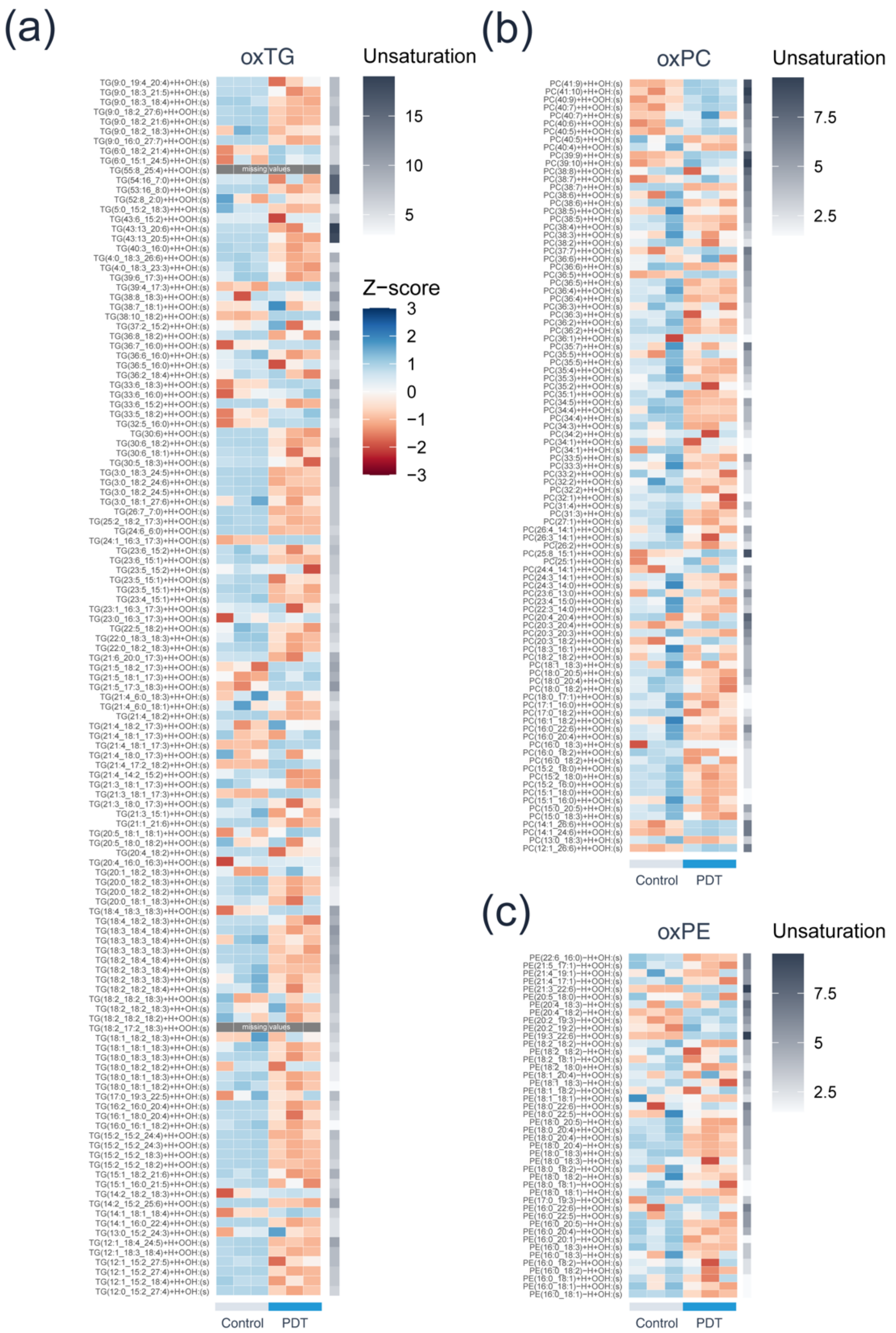

Fig. S11 Heatmaps of Z-scores for all (a) oxidized triglycerides (oxTGs), (b) oxidized phosphatidylcholines (oxPCs), and (c) oxidized phosphatidylethanolamines (oxPEs) detected using LipidSearch software (Thermo Fisher Scientific) in control and PDT-treated Intralipid samples.

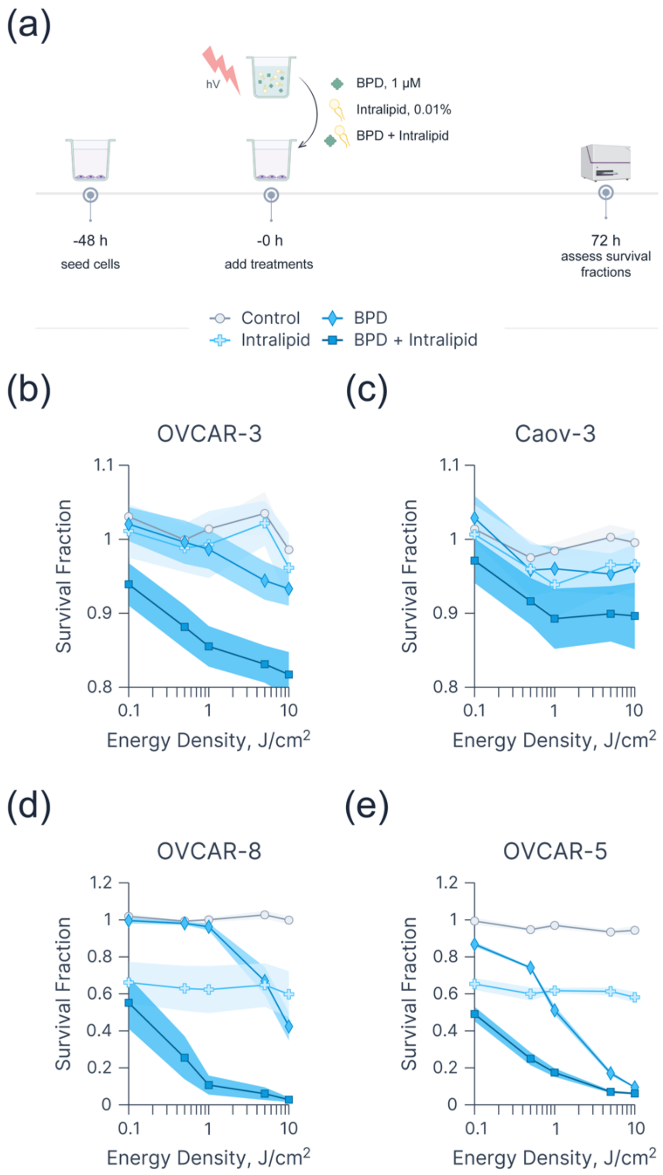

Fig. S12 Effects of BPD, Intralipid, and BPD–Intralipid mixtures exposed to 0-10 J/cm^2 on cell survival in ovarian cancer lines. **(a)** Experimental workflow schematic (created using BioRender). Survival fractions for **(b)** OVCAR-3, **(c)** Caov-3, **(d)** OVCAR-8, and **(e)** OVCAR-5 cells. Statistics: Each point represents the mean of five independent experiments, each performed in duplicate; error bars indicate the standard error of the mean.

Table S1. Differentially expressed lipid species defined as those with absolute log2 fold change > 1 (PDT/Control) and p-value < 0.05.

LipidID	Charge	CalcMz	BaseRt	Class	log2FC	pValue	log10P
**Cer(d18:0_16:0)-H**	-1	538.520468	10.4463664	Cer	1.07965791	0.00155464	2.80837106
**Cer(d18:0_22:0)-H**	-1	622.614368	12.3242542	Cer	1.20434381	0.0202328	1.69394407
**Cer(d18:0_24:0)-H+OH:(s)**	-1	666.640583	12.3491649	Cer	1.24870675	0.02616802	1.58222915
**Cer(d18:0_24:1)-H**	-1	648.630018	12.276948	Cer	1.1681267	0.0390197	1.40871612
**Cer(d18:1_23:0)-H**	-1	634.614368	12.3950398	Cer	1.14285609	0.00590863	2.22851313
**Cer(d18:2_24:1)-H+OH:(s)**	-1	660.593633	9.08455929	Cer	1.96929686	0.00684224	2.16480145
**CL(43:5_15:0_17:1)-H+OOH:(s)**	-1	1526.03304	9.16684062	CL	-3.5209938	0.00071778	3.14400595
**CL(72:4_15:0)-H+OH:(s)**	-1	1682.25722	9.14342222	CL	1.40836046	0.02797504	1.55322927
**DG(16:0_18:3)+H**	1	591.498302	8.00557923	DG	2.10823749	0.00268419	2.57118612
**DG(16:0_18:4)+H**	1	589.482652	9.96868954	DG	4.1980489	0.00126053	2.89944579
**DG(18:1_18:3)+H**	1	617.513952	8.04570876	DG	1.60983699	0.00225838	2.64620245
**DG(18:1_18:4)+H**	1	615.498302	12.8485005	DG	1.21805941	0.01109918	1.9547093
**DG(18:2_18:3)+H**	1	615.498302	7.3013025	DG	1.38593471	0.00047228	3.32579852
**DG(18:3_18:4)+H**	1	611.467002	9.12224676	DG	3.30610601	0.00290061	2.53751111
**DG(6:0_19:2)+H**	1	467.373102	8.10993877	DG	1.872373	0.00144131	2.84124214
**DG(6:0_21:3)+H**	1	493.388752	8.63221328	DG	2.19285032	0.0035194	2.4535318
**DG(6:0_21:4)+H**	1	491.373102	9.61493149	DG	2.92512858	0.00140294	2.85296214
**DG(6:0_21:5)+H**	1	489.357452	9.59506488	DG	2.79033956	0.00042685	3.36972946
**DG(O-18:4_16:0)+H**	1	575.503387	9.65102155	DG	-1.2028169	0.0327877	1.48428904
**DG(O-18:4_18:3)+H**	1	597.487737	7.42551822	DG	1.8731187	0.00049247	3.30762018
**DG(P-18:4_16:0)+H**	1	573.487737	12.8808228	DG	1.07901691	0.01294789	1.88780109
**DG(P-18:4_17:2)+H**	1	583.472087	12.8941457	DG	-1.3537675	0.01707333	1.76768167
**DG(P-18:4_18:2)+H**	1	597.487737	9.54161703	DG	1.20449667	0.0067873	2.16830298
**DG(P-18:4_18:3)+H**	1	595.472087	9.54348937	DG	3.50064393	0.00239475	2.62073954
**DG(P-18:4_18:4)+H**	1	593.456437	9.16465437	DG	3.53583644	0.00147657	2.83074501
**FA(18:1)-H**	-1	281.248604	10.0634429	FA	1.10585116	0.00433455	2.36305594
**FA(18:1)-H+CHO:(s)**	-1	295.227869	8.28740311	FA	2.33143578	0.04918969	1.30812589
**FA(18:2)-H**	-1	279.232954	9.20767396	FA	3.85327837	0.0054857	2.26076766
**FA(18:2)-H+CHO:(s)**	-1	293.212219	7.38337052	FA	5.24281229	0.02260332	1.64582776
**FA(18:3)-H**	-1	277.217304	9.06954244	FA	3.79976725	0.00620418	2.20731574
**FA(18:3)-H+CHO:(s)**	-1	291.196569	1.78596275	FA	1.2397606	0.00198374	2.70251445
**FA(20:5)-H+OH:(s)**	-1	317.212219	2.04445958	FA	1.39147996	0.02756215	1.55968698
**FA(22:0)-H**	-1	339.326854	9.25760192	FA	29.4496907	0.00801851	2.0959063
**FA(22:6)-H+CHO:(s)**	-1	341.212219	1.95793393	FA	1.24171258	0.00743125	2.12893826
**LPC(19:2)+H+OH:(s)**	1	550.350334	3.14595673	LPC	1.94403313	0.00015583	3.80733705
**LPC(28:3)+H+OOH:(s)**	1	690.470449	8.29497937	LPC	1.1267293	0.00461828	2.33551981

**LPC(28:5)+H+OOH:(s)**	1	686.439149	6.91365607	LPC	1.26401927	0.04402079	1.3563422
**LPE(22:6)-H**	-1	524.278266	8.58910829	LPE	-1.5382577	0.00278365	2.55538495
**LPE(23:7)-H+OH:(s)**	-1	552.273181	2.31148476	LPE	1.16336159	0.02044079	1.68950228
**LPE(28:3)-H+OOH:(s)**	-1	646.408946	8.49299753	LPE	1.25388623	0.00203131	2.69222347
**MG(O-25:5)+H**	1	433.367622	7.63694926	MG	1.2101191	0.00055004	3.25960888
**MG(O-25:6)+H**	1	431.351972	7.81054677	MG	2.82404973	0.00133787	2.87358643
**MG(P-18:4)+H**	1	335.258072	9.53562976	MG	-1.0534958	0.0019042	2.72028728
**PC(12:1_26:6)+H+OOH:(s)**	1	836.543614	7.29482902	PC	-1.4703567	0.00029619	3.52842611
**PC(13:0_18:3)+H+OH:(s)**	1	730.501749	6.85669089	PC	1.21096213	0.01067601	1.97159087
**PC(14:1_24:6)+H+OOH:(s)**	1	836.543614	7.63704444	PC	-1.1850587	0.00139558	2.85524447
**PC(14:1_26:6)+H+OOH:(s)**	1	864.574914	8.59662823	PC	-1.3000782	0.01956581	1.70850221
**PC(15:0_18:1)+H**	1	746.569434	9.68264447	PC	-1.4000872	0.0018971	2.72190918
**PC(15:0_18:3)+H**	1	742.538134	7.84111775	PC	1.11517189	0.00137127	2.86287835
**PC(15:0_18:3)+H+OH:(s)**	1	758.533049	7.75746268	PC	1.12889358	0.0376853	1.42382808
**PC(15:1_18:0)+H+OOH:(s)**	1	778.559264	8.39688833	PC	2.26054404	0.00179828	2.7451437
**PC(15:2_18:0)+H+OH:(s)**	1	760.548699	8.48345287	PC	1.33739765	0.01028473	1.98780725
**PC(15:2_18:0)+H+OOH:(s)**	1	776.543614	7.95256308	PC	1.62660172	0.00248726	2.60427959
**PC(16:0_18:2)+H+OOH:(s)**	1	790.559264	6.79195592	PC	1.29464401	0.04289828	1.3675601
**PC(16:0_22:4)+H**	1	810.600734	9.80554299	PC	-1.2053677	5.4395E-05	4.26444184
**PC(16:0_22:5)+H**	1	808.585084	9.55656303	PC	-1.9716389	0.00091763	3.03733286
**PC(17:0_18:2)+H**	1	772.585084	9.8173642	PC	-1.0215011	0.00080569	3.09383288
**PC(17:0_18:2)+H+OOH:(s)**	1	804.574914	6.93952001	PC	1.01596911	0.03371406	1.47218892
**PC(17:0_20:4)+H**	1	796.585084	9.65726964	PC	-1.9359296	0.00020347	3.69150386
**PC(17:1_16:0)+H+OOH:(s)**	1	778.559264	8.47106152	PC	1.55107303	0.04078683	1.38948005
**PC(17:1_18:2)+H**	1	770.569434	9.05933138	PC	-1.1719682	0.01041464	1.98235576
**PC(18:0_17:1)+H+OOH:(s)**	1	806.590564	7.76432492	PC	1.85356079	0.01427839	1.84532085
**PC(18:0_20:1)+H**	1	816.647684	11.3971937	PC	-1.2290235	0.00247176	2.60699313
**PC(18:0_20:3)+H**	1	812.616384	10.4037352	PC	-1.1635847	0.00283271	2.54779864
**PC(18:0_22:4)+H**	1	838.632034	10.5395993	PC	-1.057964	0.00259746	2.58545083
**PC(18:0_22:5)+H**	1	836.616384	9.99924152	PC	-1.0415458	0.00328602	2.48332945
**PC(18:0_22:6)+H**	1	834.600734	9.79947491	PC	-1.4652607	0.00365467	2.43715198
**PC(18:1_15:2)+H**	1	742.538134	9.67856793	PC	-1.4921996	0.02182377	1.6610702
**PC(18:3_20:4)+H**	1	804.553784	8.75360732	PC	-2.0181168	2.2536E-05	4.64712283
**PC(19:0_18:1)+H**	1	802.632034	11.1179829	PC	-1.0403408	0.03258564	1.48697375
**PC(19:0_18:2)+H**	1	800.616384	10.409066	PC	-5.3641201	2.0603E-05	4.68606496
**PC(20:3_20:4)+H+OOH:(s)**	1	864.574914	7.7045206	PC	-1.1564946	0.0008861	3.05251953
**PC(24:3_18:3)+H**	1	862.632034	9.83433574	PC	-1.3016822	0.03349968	1.47495938
**PC(26:2)+H+OOH:(s)**	1	678.434064	3.40313647	PC	3.44387055	0.02411196	1.6177675
**PC(27:1)+H+OOH:(s)**	1	694.465364	6.50278915	PC	1.00203137	0.00922093	2.03522525

**PC(31:0)+H**	1	720.553784	9.66071096	PC	-1.5015359	0.00046042	3.33684431
**PC(31:3)+H+OH:(s)**	1	730.501749	7.53950153	PC	1.03307772	0.00235711	2.62761983
**PC(32:2)+H+OH:(s)**	1	746.533049	6.37400629	PC	1.18574836	0.010964	1.96003079
**PC(33:4)+H**	1	740.522484	9.46892506	PC	-1.271767	0.0006677	3.17542053
**PC(34:2)+H+OH:(s)**	1	774.564349	6.96814181	PC	1.09067932	0.00424856	2.37175867
**PC(34:4)+H**	1	754.538134	6.19703661	PC	1.05727158	0.00066837	3.17497987
**PC(34:5)+H+OOH:(s)**	1	784.512314	5.53885475	PC	1.42663674	0.00174048	2.75933182
**PC(35:1)+H+OOH:(s)**	1	806.590564	7.85263282	PC	1.83963247	0.01529023	1.81558605
**PC(35:5)+H+OH:(s)**	1	782.533049	8.14371322	PC	1.16580087	0.00457028	2.340057
**PC(35:7)+H+OH:(s)**	1	778.501749	6.98377511	PC	1.07729538	0.03702181	1.4315424
**PC(35:7)+H+OH:(s)**	1	778.501749	6.78135976	PC	1.16191618	0.00252469	2.59779156
**PC(36:1)+H+OOH:(s)**	1	820.606214	8.18160715	PC	1.07891406	0.01300183	1.88599566
**PC(36:2)+H+OH:(s)**	1	802.595649	8.36992026	PC	1.22339256	0.00106898	2.97103068
**PC(36:3)+H+OOH:(s)**	1	816.574914	6.40987528	PC	1.04559687	0.04461183	1.35054994
**PC(36:4)+H+OH:(s)**	1	798.564349	7.43842071	PC	1.1603169	0.00286628	2.5426817
**PC(36:5)+H+OOH:(s)**	1	812.543614	6.74477437	PC	-1.4312477	0.00157316	2.8032275
**PC(37:7)+H+OOH:(s)**	1	822.527964	8.75247559	PC	-1.2524271	0.02444808	1.61175516
**PC(38:2)+H+OOH:(s)**	1	846.621864	8.30099374	PC	1.31302659	0.03538375	1.45119616
**PC(38:7)+H**	1	804.553784	6.66435874	PC	1.65649957	0.01332846	1.87521991
**PC(38:7)+H+OH:(s)**	1	820.548699	8.06290811	PC	4.5022997	0.00024559	3.6097857
**PC(38:7)+H+OOH:(s)**	1	836.543614	4.48868188	PC	1.01364682	0.00455978	2.34105637
**PC(38:8)+H**	1	802.538134	8.07739121	PC	-2.6514537	0.00507451	2.29460627
**PC(39:9)+H+OH:(s)**	1	830.533049	7.18525437	PC	-3.1045404	0.01199698	1.92092811
**PC(40:5)+H+OH:(s)**	1	852.611299	7.98457287	PC	1.06361235	0.00195049	2.70985636
**PC(40:5)+H+OOH:(s)**	1	868.606214	9.18303246	PC	-1.0264274	0.00406555	2.3908807
**PC(40:7)+H+OOH:(s)**	1	864.574914	7.9476008	PC	-1.2203921	0.01963048	1.70706907
**PC(41:10)+H+OH:(s)**	1	856.548699	7.54693325	PC	-1.6650855	0.01246433	1.90433094
**PC(41:9)+H+OH:(s)**	1	858.564349	8.28324011	PC	-1.3766637	0.00035099	3.4547114
**PC(42:8)+H**	1	858.600734	9.05394137	PC	-1.3467707	0.01377234	1.86099239
**PC(O-15:2_20:4)+H**	1	750.543219	9.73590116	PC	-2.1166479	0.00977213	2.01001074
**PC(O-18:0_18:1)+H**	1	774.637119	11.2070344	PC	-1.1334219	0.00022313	3.65144494
**PC(O-18:0_20:4)+H**	1	796.621469	10.503371	PC	-1.0780265	0.0034797	2.4584586
**PC(O-18:1_20:4)+H**	1	794.605819	9.74699816	PC	-1.7485382	0.00099255	3.00324633
**PC(O-18:2_18:3)+H**	1	766.574519	8.83221193	PC	2.97472437	0.01939193	1.71237901
**PE(16:0_18:1)-H+OH:(s)**	-1	732.518496	7.60955654	PE	1.94028843	0.00412776	2.38428584
**PE(16:0_18:2)+H**	1	716.522484	7.46562649	PE	1.22616341	0.00168781	2.77267703
**PE(16:0_18:3)+H+OH:(s)**	1	730.501749	6.85732354	PE	1.28843598	0.00947433	2.02345165
**PE(16:0_20:1)-H+OOH:(s)**	-1	776.544711	6.87922655	PE	1.3288685	0.00273642	2.56281726
**PE(16:0_22:5)+H**	1	766.538134	9.76689657	PE	-1.6031221	0.00190597	2.71988452

**PE(16:0_22:6)+H**	1	764.522484	9.19075207	PE	-1.5344472	0.01160338	1.93541561
**PE(16:1_22:6)-H**	-1	760.492281	8.59982112	PE	-1.262999	0.00017586	3.7548284
**PE(18:0_18:1)-H+OH:(s)**	-1	760.549796	8.55206325	PE	1.48942821	0.0002111	3.67552104
**PE(18:0_18:2)-H**	-1	742.539231	9.04971935	PE	-1.1695694	8.8286E-05	4.05410676
**PE(18:0_18:2)-H+OH:(s)**	-1	758.534146	8.05462445	PE	1.32276187	0.00018528	3.73216641
**PE(18:0_18:2)-H+OOH:(s)**	-1	774.529061	7.722948	PE	1.3520635	0.00607649	2.21634743
**PE(18:0_18:2)+H**	1	744.553784	8.36768405	PE	1.09493015	0.0006867	3.16323311
**PE(18:0_18:3)+H**	1	742.538134	7.84390742	PE	1.15166362	0.02081677	1.68158676
**PE(18:0_18:3)+H+OH:(s)**	1	758.533049	7.98470225	PE	1.7533	0.00591809	2.22781826
**PE(18:0_20:3)+H**	1	770.569434	10.5795388	PE	-1.0577195	0.03549319	1.449855
**PE(18:0_20:4)-H+OH:(s)**	-1	782.534146	8.56038547	PE	1.81380778	5.655E-05	4.24757038
**PE(18:0_20:4)+H+OOH:(s)**	1	800.543614	8.14455065	PE	1.02197321	0.00150509	2.82243684
**PE(18:0_22:4)+H**	1	796.585084	10.7112125	PE	-1.0187123	0.03920128	1.40669975
**PE(18:0_22:5)+H**	1	794.569434	10.4976526	PE	-1.0538089	0.04998046	1.30119978
**PE(18:0_22:6)+H**	1	792.553784	9.97827395	PE	-1.3106423	0.0105248	1.97778631
**PE(18:1_18:2)+H**	1	742.538134	9.67457321	PE	-1.5458833	0.00218136	2.66127246
**PE(18:1_20:4)+H**	1	766.538134	9.50327702	PE	-1.2993638	0.0063412	2.19782833
**PE(18:2_18:0)+H+OH:(s)**	1	760.548699	8.48222776	PE	1.21746345	0.01449329	1.83883305
**PE(19:3_22:6)-H+OOH:(s)**	-1	830.497761	9.17210363	PE	-1.2576934	7.6863E-05	4.11428243
**PE(20:4_16:0)+H**	1	740.522484	9.49567087	PE	-1.1536001	0.00335094	2.47483334
**PE(20:4_18:2)-H+OH:(s)**	-1	778.502846	8.59505502	PE	-1.394003	0.0007466	3.12691163
**PE(20:5_18:0)+H**	1	766.538134	8.15123705	PE	1.09284953	0.00088027	3.05538271
**PE(21:3_22:6)-H+OOH:(s)**	-1	858.529061	9.96978101	PE	-1.3835038	0.0006507	3.18661727
**PE(40:9)+H**	1	786.506834	9.1630849	PE	-3.0096299	0.00171686	2.7652651
**PE(O-16:1_22:6)-H**	-1	746.513016	9.57459175	PE	-3.0635294	0.02179923	1.66155879
**PE(O-18:0_22:6)+H**	1	778.574519	10.454227	PE	-1.1303432	0.02664065	1.57445518
**PE(O-18:1_18:1)-H**	-1	728.559966	11.31141	PE	-7.7119368	0.01687244	1.77282203
**PE(O-18:1_18:2)-H**	-1	726.544316	10.7627051	PE	-5.7709236	0.02413627	1.61732989
**PE(O-30:6_18:2)+H**	1	886.668419	8.81645035	PE	2.40055596	0.00073704	3.13251048
**PE(O-42:4)-H**	-1	808.622566	11.6312932	PE	-1.2505078	0.01925006	1.71556783
**PE(P-16:1_18:2)+H**	1	698.511919	9.06399345	PE	-1.5648137	6.1059E-05	4.21425335
**PE(P-16:1_22:6)+H**	1	746.511919	8.60654047	PE	-1.276228	0.02149175	1.66772827
**PE(P-18:1_20:4)+H**	1	750.543219	9.72063642	PE	-2.0742531	0.00354597	2.4502651
**PE(P-21:2_18:2)+H**	1	766.574519	9.16283692	PE	2.54673035	0.01519815	1.81820928
**PE(P-30:5_18:2)+H**	1	886.668419	8.8109882	PE	2.56642688	0.00712432	2.14725649
**PG(18:4_13:0)+H+OH:(s)**	1	717.43373	3.85179981	PG	5.8415109	0.00513108	2.28979131
**PG(20:0_8:0)+H**	1	667.454465	5.91077766	PG	1.26240887	0.0436342	1.360173
**PG(28:1_18:1)+H+OOH:(s)**	1	947.694695	10.6069597	PG	-3.4670172	0.00082823	3.08185037
**PG(O-26:1_18:3)+H**	1	869.663	8.64508567	PG	5.24893778	0.0139899	1.85418548

**PI(18:0_20:4)-H+OOH:(s)**	-1	917.539687	7.04057203	PI	1.01005582	0.01834897	1.73638836
**PI(18:2_18:0)-H+OOH:(s)**	-1	893.539687	6.86601016	PI	1.02683848	0.0061312	2.21245447
**PS(13:0_29:8)+H+OOH:(s)**	1	892.533444	4.48895411	PS	1.20738791	0.00697147	2.15667564
**PS(18:0_17:1)-H+OOH:(s)**	-1	806.518891	6.14596555	PS	2.22315867	0.00128544	2.89094983
**PS(18:2_18:2)-H**	-1	782.497761	9.65046929	PS	-1.0525281	0.00169134	2.77176968
**PS(19:2_19:2)+H+OH:(s)**	1	828.538529	5.36762975	PS	1.01054208	0.0441947	1.35462983
**PS(21:5_16:0)+H+OOH:(s)**	1	828.502144	4.72374059	PS	1.83135195	0.02560979	1.59159403
**SM(d34:0)+H**	1	705.590503	9.6838038	SM	-1.5298579	0.00349871	2.45609236
**SM(d35:1)+H**	1	717.590503	9.77504728	SM	-1.1277948	0.00013981	3.8544751
**SM(d40:1)+H**	1	787.668753	11.5066764	SM	-1.2880258	0.01055605	1.97649851
**TG(11:0_18:2_25:8)+H**	1	871.681017	9.13855687	TG	3.21983594	0.00025591	3.59191503
**TG(12:0_15:2_27:4)+H+OH:(s)**	1	895.738532	10.4775569	TG	3.62989679	0.00664112	2.17775896
**TG(12:1_15:2_18:4)+H+OH:(s)**	1	767.582032	9.52822272	TG	3.45824015	0.01697784	1.77011759
**TG(12:1_15:2_27:4)+H+OH:(s)**	1	893.722882	9.97970123	TG	3.5742144	0.00663836	2.17793949
**TG(12:1_18:3_18:4)+H+OOH:(s)**	1	823.608247	9.13017723	TG	2.8268643	0.00175186	2.75649949
**TG(12:1_18:4_24:5)+H+OOH:(s)**	1	903.670847	8.82474498	TG	3.05862057	0.00028128	3.55085636
**TG(14:1_16:0_22:4)+H+OH:(s)**	1	869.722882	10.4366045	TG	4.49276604	0.0074709	2.12662736
**TG(14:1_18:1_18:4)+H+OH:(s)**	1	839.675932	11.3519165	TG	-1.2106803	0.02133151	1.67097834
**TG(14:2_15:2_25:6)+H+OOH:(s)**	1	903.670847	6.89009738	TG	5.27169447	0.00412701	2.38436467
**TG(14:2_18:2_18:3)+H+OH:(s)**	1	837.660282	10.7071935	TG	-1.1162659	0.04632536	1.33418117
**TG(15:1_18:2_21:6)+H+OH:(s)**	1	889.691582	9.27438551	TG	4.05102836	0.03529313	1.45230977
**TG(15:2_15:2_18:2)+H+OOH:(s)**	1	827.639547	9.94733779	TG	4.31642675	0.00036727	3.4350161
**TG(15:2_15:2_18:3)+H+OOH:(s)**	1	825.623897	9.53470058	TG	3.10032116	0.00258398	2.58771004
**TG(15:2_15:2_24:3)+H+OOH:(s)**	1	909.717797	10.4675663	TG	2.96511616	0.00206922	2.68419244
**TG(15:2_15:2_24:4)+H+OOH:(s)**	1	907.702147	9.3220372	TG	3.70655578	0.00542874	2.26530072
**TG(16:0_18:3_18:3)+H**	1	851.712317	9.97276094	TG	4.03338442	0.00720069	2.1426256
**TG(16:0_18:3_18:4)+H**	1	849.696667	9.79568107	TG	3.58320604	0.00351158	2.45449738
**TG(16:2_16:0_20:4)+H+OH:(s)**	1	867.707232	9.97604209	TG	4.02445234	0.0152237	1.81747965
**TG(17:0_18:1_18:3)+H**	1	869.759267	12.5801023	TG	1.86930005	0.01089401	1.9628123
**TG(17:0_18:2_18:3)+H**	1	867.743617	12.226033	TG	1.11573965	0.00577755	2.23825636
**TG(17:2_18:2_18:3)+H**	1	863.712317	12.8912784	TG	-1.0637485	0.02986684	1.52481078
**TG(18:0_18:0_18:3)+H**	1	885.790567	13.1151442	TG	2.98006737	0.0273164	1.56357654
**TG(18:0_18:1_18:2)+H+OH:(s)**	1	901.785482	13.2448602	TG	1.57382241	0.00273929	2.56236217
**TG(18:0_18:1_18:3)+H+OH:(s)**	1	899.769832	12.8285812	TG	1.50050756	0.00180285	2.7440407
**TG(18:0_18:3_18:3)+H+OH:(s)**	1	895.738532	10.7279657	TG	3.70206952	0.01667792	1.77785823
**TG(18:1_18:1_18:3)+H**	1	881.759267	10.9677813	TG	5.05828853	0.01585783	1.79975628
**TG(18:1_18:1_18:4)+H**	1	879.743617	10.3678723	TG	4.1802137	0.00674724	2.17087385
**TG(18:1_18:3_18:4)+H**	1	875.712317	13.075024	TG	-1.3004247	0.01662618	1.77920751
**TG(18:2_16:0_18:4)+H**	1	851.712317	10.150017	TG	4.55088358	0.01072104	1.96976292

**TG(18:2_18:2_18:2)+H+OOH:(s)**	1	911.733447	11.4713865	TG	2.39746325	0.02256931	1.64648166
**TG(18:2_18:2_18:3)+H**	1	877.727967	9.84583726	TG	3.58763703	0.01074129	1.96894363
**TG(18:2_18:2_18:3)+H+OH:(s)**	1	893.722882	11.0228356	TG	2.08008771	0.0017956	2.7457915
**TG(18:2_18:2_18:4)+H**	1	875.712317	10.9811995	TG	2.97345315	0.01455875	1.83687579
**TG(18:2_18:2_18:4)+H+OH:(s)**	1	891.707232	9.60412432	TG	3.01531055	0.0043909	2.35744634
**TG(18:2_18:3_18:3)+H+OH:(s)**	1	891.707232	11.138145	TG	1.03654011	0.02781664	1.55569535
**TG(18:2_18:3_18:3)+H+OH:(s)**	1	891.707232	7.79261936	TG	6.01867323	0.01828301	1.73795237
**TG(18:2_18:3_18:3)+H+OH:(s)**	1	891.707232	7.72898862	TG	7.8468691	0.01156066	1.93701728
**TG(18:2_18:3_18:4)+H**	1	873.696667	9.11640424	TG	3.37282209	0.00026177	3.58207775
**TG(18:2_18:4_18:4)+H+OH:(s)**	1	887.675932	8.81429633	TG	3.3686837	0.00265487	2.57595656
**TG(18:3_18:2)+H**	1	629.477567	6.83364875	TG	1.47576916	0.00070902	3.14933942
**TG(18:3_18:3_18:3)+H+OH:(s)**	1	889.691582	7.32552806	TG	5.9050619	0.01888808	1.72381216
**TG(18:3_18:3_18:4)+H**	1	871.681017	6.87951396	TG	7.16418755	0.01221329	1.91316718
**TG(18:3_18:4_18:4)+H+OH:(s)**	1	885.660282	8.30487151	TG	3.05432464	0.00785897	2.10463416
**TG(18:4_18:3_18:3)+H+OOH:(s)**	1	903.670847	8.69974836	TG	3.69194955	0.0261646	1.58228585
**TG(20:0_18:2_18:2)+H+OH:(s)**	1	927.801132	13.2661599	TG	2.46309898	0.00362592	2.44058201
**TG(20:0_18:2_18:3)+H+OH:(s)**	1	925.785482	12.9012668	TG	1.3275308	0.00099151	3.00370367
**TG(21:1_21:6)+H+OH:(s)**	1	725.535082	9.46226987	TG	2.36823579	0.0144038	1.84152285
**TG(21:4_14:2_15:2)+H+OOH:(s)**	1	851.639547	8.44034246	TG	1.83317069	0.00083509	3.07826863
**TG(21:4_17:2_18:2)+H+OH:(s)**	1	919.738532	11.8403389	TG	-2.2890401	0.0015171	2.81898578
**TG(21:4_18:1_17:3)+H+OH:(s)**	1	919.738532	12.5377879	TG	-1.8722052	1.3983E-05	4.85441158
**TG(21:4_18:2)+H+OH:(s)**	1	685.503782	9.5489951	TG	3.13196054	0.00034258	3.46523566
**TG(21:6_3:0_6:0)+H**	1	543.368017	9.14496583	TG	2.69305868	0.00429576	2.36696003
**TG(22:0_18:1_18:4)+H**	1	937.821867	13.602491	TG	19.5946784	0.00786141	2.10449957
**TG(22:0_18:3_18:3)+H+OH:(s)**	1	951.801132	12.9294073	TG	1.17653817	0.01021637	1.99070348
**TG(23:5_15:1)+H+OOH:(s)**	1	687.483047	4.45964496	TG	1.90545346	0.03517505	1.45376522
**TG(23:6_15:1)+H+OOH:(s)**	1	685.467397	4.42848942	TG	1.93384445	0.00011076	3.95563407
**TG(23:6_15:2_18:3)+H**	1	897.696667	12.6858053	TG	-2.3537443	0.01576382	1.80233849
**TG(23:6_15:2)+H+OH:(s)**	1	667.456832	6.04911948	TG	1.02489907	0.01695241	1.77076848
**TG(24:1_16:3_17:3)+H+OOH:(s)**	1	951.764747	11.5532581	TG	-31.455585	0.00399136	2.39887946
**TG(24:6_6:0)+H+OOH:(s)**	1	575.357847	4.45268312	TG	3.41227091	0.00014273	3.84548111
**TG(25:2_18:2_17:3)+H+OOH:(s)**	1	993.811697	13.2785552	TG	2.90357726	0.00181469	2.7411982
**TG(25:5_5:0_6:0)+H**	1	629.477567	10.6288628	TG	2.31725302	0.00415363	2.38157204
**TG(26:7_7:0)+H+OOH:(s)**	1	615.389147	4.41293376	TG	3.67112752	0.00114907	2.9396547
**TG(3:0_15:2_18:2)+H**	1	631.493217	7.41583808	TG	1.5138507	0.01620409	1.7903754
**TG(3:0_15:2_18:4)+H**	1	627.461917	9.62534356	TG	3.0446899	0.01607584	1.79382641
**TG(3:0_16:0_27:6)+H**	1	767.618417	9.9582395	TG	5.15304564	0.01462894	1.834787
**TG(3:0_18:1_24:4)+H**	1	755.618417	12.3316444	TG	1.70075174	0.00306933	2.51295643
**TG(3:0_18:1_27:6)+H**	1	793.634067	9.9681753	TG	3.79015507	0.0025669	2.59059036

**TG(3:0_18:1_27:6)+H+OH:(s)**	1	809.628982	9.51034665	TG	3.38354162	0.00193964	2.71227791
**TG(3:0_18:2_24:4)+H**	1	753.602767	11.9389929	TG	1.44404338	0.00039494	3.40346895
**TG(3:0_18:2_24:5)+H**	1	751.587117	9.58595848	TG	5.10068457	0.00440892	2.35566749
**TG(3:0_18:2_24:5)+H+OH:(s)**	1	767.582032	9.63250469	TG	2.7123285	0.00206695	2.68467033
**TG(3:0_18:2_24:6)+H+OH:(s)**	1	765.566382	9.08980505	TG	4.54077041	0.00236716	2.62577172
**TG(3:0_18:2_26:4)+H**	1	781.634067	11.0845254	TG	2.68251946	0.00128529	2.89099894
**TG(3:0_18:2_26:5)+H**	1	779.618417	11.1240538	TG	1.73782511	0.000327	3.48544727
**TG(3:0_18:2_26:5)+H**	1	779.618417	9.54988663	TG	3.64140822	0.00434269	2.36224091
**TG(3:0_18:2_26:5)+H**	1	779.618417	9.72312451	TG	3.85601934	0.04784159	1.32019443
**TG(3:0_18:2_27:6)+H**	1	791.618417	9.51744057	TG	3.01140941	0.00598472	2.22295599
**TG(3:0_18:2_27:7)+H**	1	789.602767	11.1514693	TG	1.16315588	0.04156653	1.38125625
**TG(3:0_18:2_27:7)+H**	1	789.602767	9.12457207	TG	3.06190699	0.00161791	2.79104638
**TG(3:0_18:2_30:7)+H**	1	831.649717	9.11989875	TG	3.41312031	0.00362556	2.44062486
**TG(3:0_18:2_30:8)+H**	1	829.634067	8.80563656	TG	3.78973769	0.00769594	2.11373832
**TG(3:0_18:3_24:5)+H+OH:(s)**	1	765.566382	9.27119614	TG	5.02273923	0.00222717	2.65224591
**TG(3:0_18:3_26:5)+H**	1	777.602767	9.5435298	TG	2.89373045	0.0012441	2.90514596
**TG(3:0_18:3_27:6)+H**	1	789.602767	7.36806648	TG	9.55259902	0.03534475	1.45167508
**TG(3:0_18:3_27:7)+H**	1	787.587117	8.71029651	TG	4.55314184	0.00310154	2.5084224
**TG(3:0_6:0_18:4)+H**	1	505.352367	9.64242035	TG	3.17031983	0.00248554	2.60457979
**TG(3:0_6:0_21:5)+H**	1	545.383667	9.55672681	TG	3.46411226	0.00519484	2.28442753
**TG(3:0_6:0_27:5)+H**	1	629.477567	4.48030284	TG	2.69678797	0.00279605	2.55345566
**TG(30:4_25:4_25:5)+H**	1	1230.04097	17.0016944	TG	2.09805215	0.00993059	2.00302488
**TG(30:6_18:2)+H+OH:(s)**	1	807.613332	8.8076046	TG	7.40523363	0.01385675	1.85833867
**TG(32:5_16:0)+H+OH:(s)**	1	813.660282	11.3575605	TG	-1.1055093	0.04282224	1.36833057
**TG(33:5_18:2)+H+OOH:(s)**	1	867.670847	10.9124092	TG	-1.1298182	0.02935374	1.53233663
**TG(33:6_15:2)+H+OOH:(s)**	1	823.608247	7.40916176	TG	5.0995989	0.02449838	1.61086268
**TG(33:6_18:3)+H+OOH:(s)**	1	863.639547	9.84946732	TG	-1.3900012	0.0306507	1.51355954
**TG(36:8_18:2)+H+OOH:(s)**	1	903.670847	8.12273924	TG	1.86283801	0.04147744	1.38218805
**TG(38:10_18:2)+H+OOH:(s)**	1	927.670847	10.4108511	TG	-3.0026798	0.00173409	2.7609272
**TG(38:4_22:5)+H**	1	957.790567	12.0605057	TG	3.04152606	3.9675E-05	4.40148832
**TG(39:4_17:3)+H+OH:(s)**	1	921.754182	12.2796301	TG	-2.2784196	0.00313476	2.5037957
**TG(39:6_17:3)+H+OOH:(s)**	1	933.717797	10.8936563	TG	2.5638966	0.00416176	2.38072278
**TG(4:0_15:2_17:3)+H**	1	629.477567	10.4136375	TG	2.89369103	0.00073345	3.13462826
**TG(4:0_18:3_26:6)+H+OOH:(s)**	1	821.592597	8.65070182	TG	3.28562284	0.00256861	2.59030182
**TG(40:3_16:0)+H+OH:(s)**	1	929.816782	13.5959962	TG	2.73474935	0.0100815	1.99647475
**TG(5:0_15:2_18:3)+H+OH:(s)**	1	673.503782	7.78294305	TG	1.01646755	0.01517135	1.81897577
**TG(51:9_28:4)+H**	1	1216.02532	16.7213854	TG	6.63782761	0.00976027	2.01053802
**TG(53:16_8:0)+H+OH:(s)**	1	973.691582	8.84052325	TG	4.74913511	0.00794119	2.10011465
**TG(6:0_15:1_24:5)+H+OH:(s)**	1	769.597682	9.63220397	TG	1.19264508	0.00924356	2.03416061

**TG(6:0_18:2_21:4)+H+OH:(s)**	1	769.597682	12.0469159	TG	-1.6389748	0.0141194	1.85018384
**TG(63:19)+H**	1	979.681017	8.4573798	TG	2.16315734	0.00056277	3.24966799
**TG(7:0_18:0_29:5)+H**	1	881.759267	10.9151354	TG	4.89906757	0.011943	1.92288659
**TG(9:0_16:0_27:7)+H+OOH:(s)**	1	881.686497	9.76065415	TG	2.98846603	0.00823087	2.08455433
**TG(9:0_18:2_21:6)+H+OH:(s)**	1	807.613332	9.52214421	TG	3.33277858	0.00196499	2.7066406
**TG(9:0_18:2_27:6)+H+OOH:(s)**	1	907.702147	10.0860763	TG	2.35018635	0.00022902	3.64012242
**TG(9:0_18:3_18:4)+H+OH:(s)**	1	767.582032	7.35305597	TG	4.46231623	0.01462839	1.83480343
**TG(9:0_18:4_27:5)+H**	1	873.696667	7.29046228	TG	7.4644457	0.01301129	1.88567971
**TG(O-10:0_6:0_20:6)+H**	1	613.482652	7.34745426	TG	1.6509675	0.00024557	3.60982386
**TG(O-15:1_18:3_21:3)+H**	1	863.748702	12.337232	TG	1.37752184	0.02211679	1.65527787
**TG(O-15:1_3:0_18:3)+H**	1	617.513952	8.00538678	TG	1.55912167	0.00340579	2.4677822
**TG(O-15:1_3:0_18:4)+H**	1	615.498302	7.53626113	TG	2.05642499	0.00253478	2.59605963
**TG(O-15:2_3:0_18:4)+H**	1	613.482652	4.48151349	TG	3.03959945	0.00183405	2.73658874
**TG(O-22:6_3:0_5:0)+H**	1	529.388752	9.53973293	TG	2.98171284	0.00059094	3.22845856
**TG(O-27:8_3:0_6:0)+H**	1	609.451352	9.25003532	TG	2.57470337	0.01236674	1.90774481
**TG(O-28:8_3:0_5:0)+H**	1	609.451352	9.70324508	TG	2.70164478	0.0053868	2.26866903
**TG(O-63:18)+H**	1	967.717402	9.84840174	TG	3.20819536	0.02145274	1.66851731
**TG(O-63:19)+H**	1	965.701752	8.90117646	TG	4.47501866	0.03519491	1.45352009
**TG(P-15:2_3:0_18:2)+H**	1	615.498302	10.3751593	TG	3.02279914	0.00039207	3.40663571
**TG(P-15:2_3:0_18:4)+H**	1	611.467002	9.15350874	TG	3.28793228	0.00229695	2.63884807
**TG(P-18:4_3:0_6:0)+H**	1	489.357452	9.60761419	TG	2.76039507	0.00150736	2.82178284
**TG(P-4:0_3:0_23:4)+H**	1	531.404402	7.2911185	TG	1.81951463	2.688E-05	4.5705652
**TG(P-4:0_3:0_23:5)+H**	1	529.388752	9.53332183	TG	3.05852861	0.00044225	3.35432864
**TG(P-46:15_15:2)+H**	1	939.686102	9.74684891	TG	2.29372723	0.00402725	2.39499124
**TG(P-49:16_12:0)+H**	1	941.701752	9.95318453	TG	4.11884436	0.00281137	2.55108147
**TG(P-5:0_18:4_13:1)+H**	1	613.482652	7.64768822	TG	2.38483884	0.01476114	1.83088004
**TG(P-6:0_12:1_18:3)+H**	1	615.498302	9.95402226	TG	2.81193106	0.00244092	2.61244614

Table S2. IC50s and R-squared values for Extracellular-PDT-treated cells, in which Intralipid and BPD were taken out immediately post light exposure. These and other R-squared values and IC50s (J/cm^2) were calculated in Graphmatik.io.

	R-squared		IC50	
Intralipid, %	0	0.01	0%	0.01
OVCAR-3	0.99	0.97	0.26	0.45
OVCAR-5	0.99	0.99	0.08	0.12
OVCAR-8	0.98	0.99	0.06	0.12
Caov-3	0.99	0.99	0.18	0.26

Table S3. IC50s and R-squared values for Extracellular-PDT-treated cells, in which light-exposed BPD and Intralipid and BPD were left in wells for 72 h. These and other R-squared values and IC50s (J/cm^2) were calculated in Graphmatik.io.

	R-squared		IC50	
Intralipid, %	0	0.01	0	0.01
OVCAR-3	0.99	0.97	0.22	0.28
OVCAR-5	0.99	0.99	0.07	0.12
OVCAR-8	0.99	0.99	0.05	0.08
Caov-3	0.98	0.98	0.16	0.31

Table S4. IC50s and R-squared values for Intracellular-PDT-treated cells, in which Intralipid was taken out immediately post light exposure. These and other R-squared values and IC50s (J/cm^2) were calculated in Graphmatik.io.

	R-squared		IC50	
Intralipid, %	0	0.01	0	0.01
OVCAR-3	0.99	0.98	0.19	0.19
OVCAR-5	0.99	0.99	0.17	0.13
OVCAR-8	0.98	0.98	0.1	0.12
Caov-3	0.99	0.99	0.17	0.16

Table S5. IC50s and R-squared values for Intracellular-PDT-treated cells, in which light-exposed Intralipid was left in wells for 72 h. These and other R-squared values and IC50s (J/cm^2) were calculated in Graphmatik.io.

	R-squared		IC50	
Intralipid, %	0	0.01	0	0.01
OVCAR-3	0.99	0.98	0.16	0.14
OVCAR-5	0.99	0.99	0.14	0.12
OVCAR-8	0.99	0.99	0.09	0.07
Caov-3	0.99	0.99	0.13	0.12
